# Engineering of induced pluripotent stem cells for the efficient development of non-alloreactive, hypoimmunogenic CD8αβ CAR-T cells

**DOI:** 10.3389/fimmu.2026.1757174

**Published:** 2026-02-20

**Authors:** Alexandros Nianias, Afroditi Katsarou, Henk Jan Prins, Aida Shahrabi, Cindy van Velzen, Linda Henneman, Ruud Ruiter, Tuna Mutis, Richard Groen, Maria Themeli

**Affiliations:** 1 Amsterdam University Medical Centers (UMC) location Vrije Universiteit Amsterdam, Departement of Hematology, Amsterdam, Netherlands; 2 Cancer Center Amsterdam, Cancer Biology and Immunology, Amsterdam, Netherlands; 3 Animal Modeling Facility, Netherlands Cancer Institute, Amsterdam, Netherlands

**Keywords:** allogeneic, CAR-T cell therapy, chimeric antigen receptor, hypoimmunogenic, induced pluripotent stem cells

## Abstract

**Background:**

Induced pluripotent stem cells (iPSCs) are a promising platform to produce “off-the-shelf” chimeric antigen receptor (CAR)-engineered T cells (CAR-T) with thepotential for multiplex genetic engineering.

**Methods:**

Here, we employed genome editing to knock out the TRAC and B2M genes in iPSC lines, while simultaneously harnessing the edited loci to introduce a drug-inducible CAR and the human leukocyte antigen (HLA)-E single chain trimer.

**Results:**

The inducible CAR expression allowed the robust generation of T-cell receptor (TCR)-negative CD8ab+ CAR-T cells with demonstrable anti-tumor efficacy and lack of alloreactivity. HLA-class Inegative, HLA-Epositive CD8 CAR-T cells were protected against immune rejection; however, disruption of B2M resulted in genomic instability and affected the efficiency of T-cell development and the functionality of the generated T cells.

**Discussion:**

Facilitating multiplex engineering at well-characterized genomic loci and regulation of possible developmental effects will empower the use of engineered iPSC as a viable method to efficiently produce “off-the-shelf” CART cells.

## Introduction

The clinical success of cell therapy with chimeric antigen receptor (CAR)-engineered T (CAR T) cells against hematological malignancies opens up new avenues for the further exploitation of this breakthrough therapy for more patients and additional diseases. Unfortunately, the rapid development of CAR-T cell therapy has also revealed important challenges that limit the feasibility of cost-effective, easier, and broader application. Current CAR-T cell manufacturing is time-consuming and the required vein-to-vein processing time can be critical in cases of rapid disease progression. Furthermore, the isolation, genetic modification, and expansion of autologous T cells is, in many cases, technically challenging and leads sometimes to production failure (1). In addition, the quality of the patient-derived, apheresis product is highly variable, as it depends on the type and stage of the disease, previous therapies, and immune cell composition (1–3). The use of allogeneic healthy donor-derived T cells can be an alternative, readily available source of CAR-T cells. However, their use requires substantial genome engineering to avoid graft-versus-host disease (GvHD) and graft rejection (4–6). Ensuring sufficient production yield of safe, primary allogeneic CAR-T cells with consistent functional properties after multiplex genetic engineering is still challenging (7).

To overcome the aforementioned issues, induced pluripotent stem cells (iPSCs) have been proposed as an alternative and perpetual source of therapeutic CAR-T cells (8). iPSC’s unlimited self-renewal capacity allows for long-term use and theoretically endless genetic modifications in order to accommodate all the desirable characteristics for their T-cell derivatives (iT), such as tumor specificity, improved efficacy, and reduced immunogenicity. In contrast to primary T cells, fully modified clonal iPSC lines can be selected and extensively evaluated, resulting in a stable, scalable, and safe source of CAR-T cells (9).

Previously, T cell-derived iPSCs (T-iPSCs) were engineered to express a CD19-targeting CAR and differentiated to functional CD19-specific T-iPSC-derived CAR-T cells (CAR-iT) (10). However, phenotypic, functional, and transcriptional analysis revealed that the CAR-iT cells were rather CD8αβ^−^CD4^−^ double-negative (DN) or CD8αα single-positive (SP), resembling more to innate cells than mature CD8 or CD4 T cells (10, 11). According to the physiological model of T-cell development, a critical step to obtain mature CD8αβ^+^ T cells is the process of β-selection and the emergence of CD4^+^CD8αβ^+^ double-positive (DP) cells (12). Recently, van der Stegen et al. showed that the lineage skewing in CAR-iT cells can be attributed to premature expression of the endogenous TCR as well as the constitutively expressed CAR (13). The efficient transition of CAR-engineered T-iPSC-derived lymphoid progenitors to β-selection is partially restored by the absence of a TCR, the timely CAR expression driven by the endogenous TCRα promoter, and a calibrated CAR signaling (13).

To ensure the broad applicability of CAR-iT cells, genetic modifications need to be applied to guarantee their safe use on an allogeneic basis. Apart from facilitating the development of mature CAR-iT cells, TCR silencing warrants the absence of graft-versus-host reactivity. Furthermore, hypoimmunogenic pluripotent stem cell (PSC) lines can be generated by the elimination of HLA class I and II expression through the disruption of *B2M* and *CIITA* genes, respectively (14). Additionally, introduction of non-classical HLA class I molecules (HLA-E and HLA-G) or patient-specific HLA-C and genetic knockout of NK cell activating ligands (CD155, B7-H3) have been shown to reduce NK-mediated rejection of iPSC-derived cellular products (15–17).

In this study, we used CRISPR-mediated genome editing to engineer TCR^negative^ and HLA class I^negative^ T-iPSC lines by knocking out the *TRAC* and *β2m* genes. The 4 alleles of these edited loci were successfully harnessed to introduce transgenes of interest, such as an inducible CD38-CAR expression cassette and an HLA-E single chain trimer (HLA-E SCT) (18). We demonstrate that expression of the CAR at a more “physiological” developmental stage can overcome the issues of misplaced CAR signaling and allow the efficient generation of non-alloreactive CD38CAR-iT cells, which successfully controlled tumor growth *in vivo*. Disruption of *B2M* and HLA-E SCT insertion not only protected CAR-iT against T cell- and NK cell-mediated rejection, but also led to the expansion of chromosomally unstable T-iPSCs, the decreased efficiency of iT cell development, and the reduced expansion of CAR-iT cells.

## Results

### Generation of *TCRα*^−/−^ T-iPSC clones engineered with an inducible CAR

**Figure 1:**
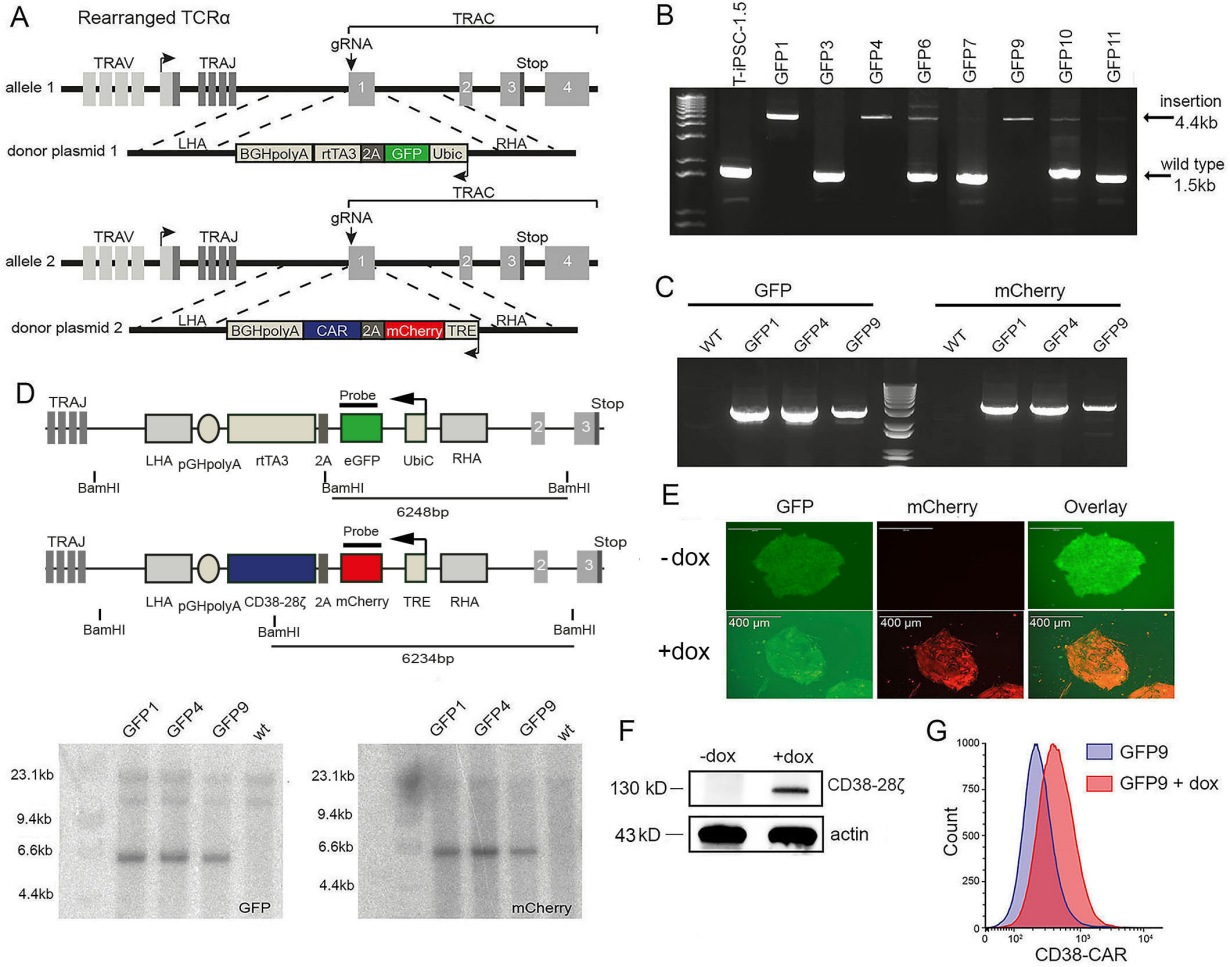
Generation of TCRα^−/−^T-iPSC clones bearing an inducible CAR. **(A)** Schematic strategy of CRISPR/Cas9-mediated bi-allelic disruption of the TRAC locus and insertion of indCD38CAR by homologous recombination. Arrows show the position of the double-strand break at the beginning of exon 1. **(B)** PCR for verification of bi-allelic donor plasmid integration. Arrows indicate the size of the insert template or of the original sequence. **(C)** Confirmation by PCR of the insertion of the GFP and the mCherry carrying donor cassettes. **(D)** Southern blot analysis for target-specific integration of donor templates using molecular probes for GFP and mCherry sequences after digestion with BamHI **(E)** Representative fluorescence microscopy images showing expression of GFP and mCherry of T-iPSC 1.5 GFP9 colonies before and after dox treatment for 48 h. Scale bar, 400 μm. **(F)** Western blot for CAR detection upon dox treatment on GFP9 T-iPSC clone. Actin was used as reference gene. **(G)** Detection of the CD38CAR surface expression after the addition of dox on GFP9 T-iPSC by staining with an anti-human F(ab′)_2_ antibody.

In order to facilitate the development of mature CAR-iT cells and ensure the absence of alloreactivity, we used CRISPR to knock out the *TCRα* chain by targeting the exon 1 of the *TRAC* locus of an established T-iPSC line with demonstrated potential to generate iT cells *in vitro* (T-iPSC 1.5, **Table 1**) (10) (**Figure 1A**). We hypothesized that temporal induction of CAR expression in the DP cells will facilitate efficient production of mature CD8αβ^+^ T cells. To investigate this, we used a doxycycline (dox)-inducible Tet-On system to control the expression of a second-generation CD38-CAR (19). We used homologous recombination and simultaneously inserted the elements of the Tet-On inducible CAR (indCAR) expression (**Figure 1A**). Briefly, one template encoded the constitutive expression of green fluorescent protein (GFP) and reverse tetracycline-controlled transactivator (rtTA) and the other template encoded the doxycycline-inducible expression of mCherry and the CD38-CAR. Eleven T-iPSC clones were selected based on GFP expression (clones GFP1-11), eight of which were further assessed by PCR for bi-allelic targeting of the *TRAC* locus (**Table 2**). Three of these clones, GFP1, GFP4, and GFP9 (**Table 1**), were verified to be targeted in both alleles with an insertion (**Figure 1B** and **Supplementary Figure S1**) and contained both the GFP and mCherry transgenes (**Figure 1C** and **Supplementary Figure S2**), indicating that each template has been inserted in a separate *TRAC* allele. Southern blot analysis confirmed a single copy insertion of every template in each of the 2 alleles of the *TRAC* locus (**Figure 1D** and **Supplementary Figures S3A, B**). After treatment with dox, the induction of mCherry expression (**Figure 1E**) and the indCAR expression were confirmed in protein level (**Figures 1F, G** and **Supplementary Figure S4**). TCRα^−/−^ indCAR T-iPSC displayed an intact chromosomal pattern (**Supplementary Figure S5**). Using the same strategy, we also generated *TCRα*^−/−^ T-iPSC with an inducible CD19-CAR (data not shown).

**Table 1:** Names of iPSC lines and passages at the time of generation.

iPSC line	Work passage number
1.5	22
1.5 GFP1	33
1.5 GFP4	32
1.5 GFP9	31
1.5 GFP9BFP2.1	47
1.5 GFP9BFP2.3	47
1.5 BFP2.1	31
1.5 B2MKO	31
1.5–19 G8	43

**Table 2:** Oligonucleotide sequences used for genome editing and molecular verification.

Oligonucleotide	Sequence
TRAC gRNA	5′-CAGGGTTCTGGATATCTGTGGG-3′
β2m gRNA	5′-GGCCGAGATGTCTCGCTCCGTGG-3′
CD38 gRNA	5′-TCAGACCGTACCTTGCAACA-3′
TRAC primer 1	5′-CAGCAAAGGAACTATGCCTTAG-3′
TRAC primer 2	5′-CAGCTGGATGTCTGCATTGC-3′
GFP primer 1	5′-GTGAGCAAGGGCGAGGAGCT-3′
GFP primer 2	5′-AACGGCCACAAGTTCAGCGTG-3′
mCherry primer 1	5′-TGAGCAAGGGCGAGGAGG-3′
mCherry primer 2	5′-CATGCGCTTCAAGGTGCACA-3′
CD38 CAR-E2A-mCherry cDNA primer 1	5′-ACGCTTTGTTGAAACTCGCTGGCGATGTTGAAAGTAACCCCGGTCCCATGGCTCTCCCAGTGACT-3′
CD38 CAR-E2A-mCherry cDNA primer 2	5′-GCGGCCGCGCTAGCTTAGCGAGGGGGCAGGGCCTGCAT-3′
BFP primer 1	5′-TCCATCGCCAccATGGTGAGCAAGGGCGAGGA-3′
BFP primer 2	5′-CTTGTACAGCTCGTCCATGC-3′
β2m primer 1	5′-AGTTATTGAGAGGGCTGGT-3′
β2m primer 2	5′-GTCGCCTAGGTTCTGATGATG-3′
β2m primer 3	5′-GGACTCCACCACCACGAAAT-3′
β2m primer 4	5′-TTATCTATGGCGGAAGATAACTG-3′
GFP probe for Southern blot	5′-GTGGCTGTTGTAGTTGTACTCCAGCTTGTGCCCCAGGATGTTGCCGTCCTCCTTGAAGTCGATGCCCTTCAGCTCGATGCGGTTCACCAGGGTGTCGCCCTCGAACTTCACCTCGGCGCGGGTCTTGTAGTTGCCGTCGTCCTTGAAGAAGATGGTGCGCTCCTGGACGTAGCCTTCGGGCATGGCGGACTTGAAGAAGTCGTGCTGCTTCATGTGGTCGGGGTAGCGGCTGAAGCACTGCACGCCGTAGGTCAGGGTGGTCAC-3′
mCherry Probe for Southern blot	5′-CTGCACGGGCTTCTTGGCCTTGTAGGTGGTCTTGACCTCAGCGTCGTAGTGGCCGCCGTCCTTCAGCTTCAGCCTCTGCTTGATCTCGCCCTTCAGGGCGCCGTCCTCGGGGTACATCCGCTCGGAGGAGGCCTCCCAGCCCATGGTCTTCTTCTGCATTACGGGGCCGTCGGAGGGGAAGTTGGTGCCGCGCAGCTTCACCTTGTAGATGAACTCGCCGTCCTGCAGGGAGGAGTCCTGGGTCACGGTCACCACGCCGCCGTCCTCGAAGTTCATCACGCGCTCCCACTTGAAGCCCTC-3′
BFP probe for Southern blot	5′-ACCATGTGATCGCGCTTCTCGTTGGGGTCTTTGCTCAGCACGGACTGGGTGCTCAGGTAGTGGCTGTCGGGCAGCAGCACGGGGCCGTCGCCGATGGGGGTGTTCTGCTGGTAGTGGTCGGCGAGCTGCACGCTGCCGTCCTCCACGTTGTGGCGGATCTTGAAGTTCACCTTGATGCCGTTCTTCTGCTTGACGGCCATGATATAGATGTTGTGGCTGTTGAAGTTGTACTCCAGCTTGTGCCCCAGGATGTTGCCGTCCTCCTTGAAGTCGACGCCCTTCAGCTCGATGCGGTTCACC-3′

### *TCRα*^−/−^ indCAR T-iPSC efficiently generate mature and functional TCR^neg^CD8αβ^+^ CAR-iT cells

**Figure 2:**
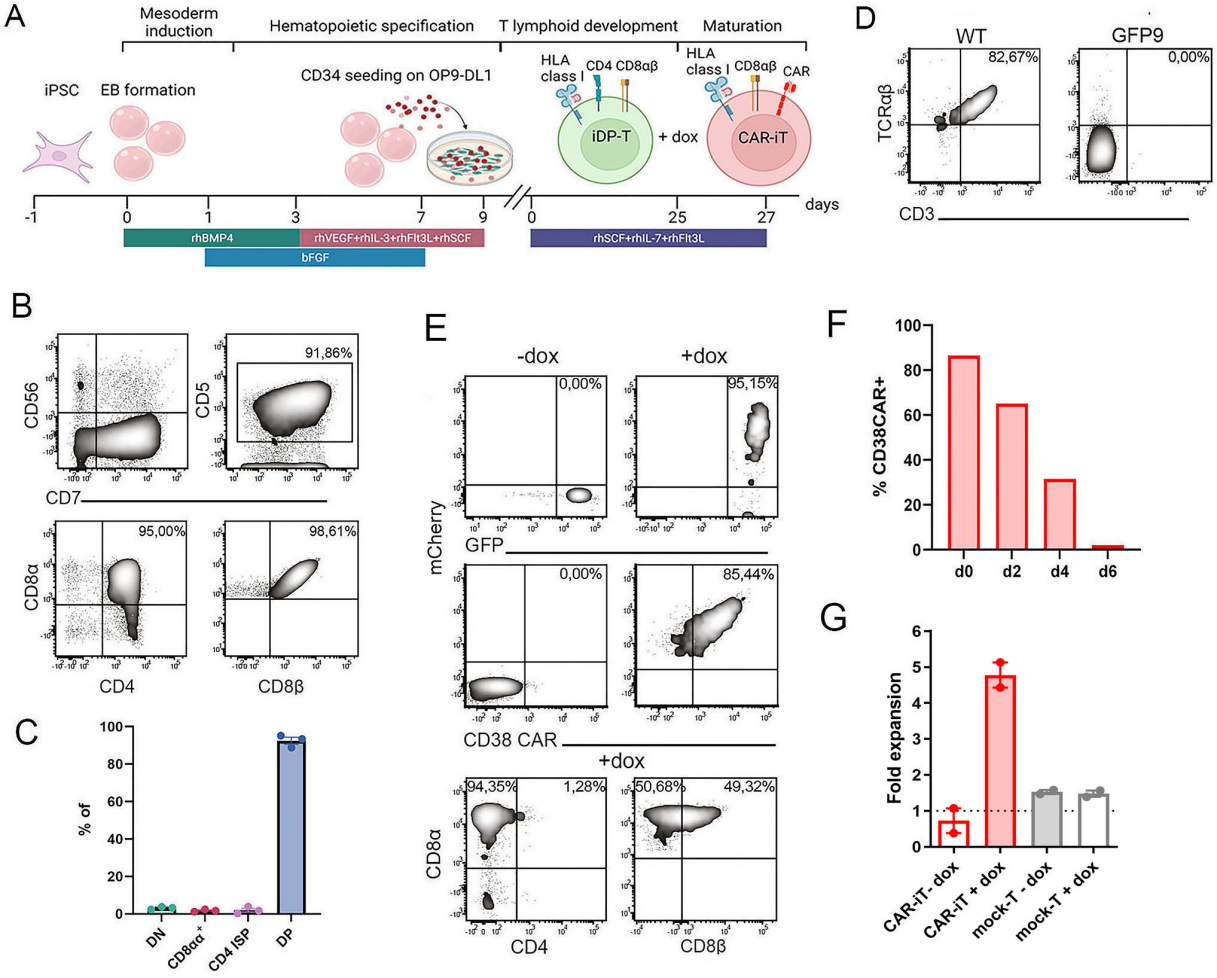
Phenotypic and functional assessment of TCR^neg^indCD38CAR-iT cells. **(A)** Schematic representation of T-cell differentiation protocol. T-iPSCs give rise to CD34^+^ hematopoietic progenitors via EB formation (day 0 to day 9). Subsequent co-culture with OP9-DL1 T cells induces T-cell commitment and generation of CD4^+^CD8αβ^+^ DP thymocytes (Tdiff day 0 to day 25). The addition of dox for 48 h leads to CAR expression and maturation into CD8αβ^+^ CAR-iT cells (Tdiff day 27). **(B)** Representative flow cytometry plots of GFP9 CD4^+^CD8αβ^+^ DP thymocytes at Tdiff day 25 for expression of T cell-specific markers. **(C)** Phenotype distribution of TCR^neg^indCD38CAR-iT cells on day 25 of T-cell commitment (*n* = 3 independent differentiations). Data are presented as mean ± SEM. **(D)** Immunophenotypic validation of CRISPR/Cas9-mediated TCRα disruption at Tdiff day 25. **(E)** Representative flow cytometry plots upon dox treatment for 48 h on GFP9 DP thymocytes at Tdiff day 27. mCherry levels correlate with CAR expression after staining with an anti-human F(ab′)_2_ antibody in a linear fashion. Emergence of CAR on the cell surface of GFP9 DP thymocytes leads to maturation into CD8αβ^+^ CAR-iT cells. **(F)** Kinetics of CD38 CAR surface expression upon dox withdrawal by staining with anti-human F(ab′)_2_ antibody. **(G)** Expansion of TCR^neg^ indCD38CAR-iT cells in the absence (clone 1.5 GFP9, *n* = 2 independent differentiations) and in the presence of dox (clone 1.5 GFP4, *n* = 1 and clone 1.5 GFP9, *n* = 1) as well as TRE-mock transduced primary T cells with and without dox treatment (*n* = 2 donors) after co-culture on irradiated allogeneic feeder mix. Data are presented as mean ± SEM. Panel **(A)** was Created in BioRender. Themeli, M. (2026) https://BioRender.com/b2b1eol.

We further sought to investigate if the temporal control of CAR expression would lead to the successful generation of DP thymocytes and SP iT cells. *TCRα*^−/−^ indCAR-T-iPSCs were differentiated towards the lymphoid lineage using a previously described modified protocol (**Figure 2A**) (10). Indeed, in the absence of CAR expression, *TCRα*^−/−^ indCAR-T-iPSC-derived cells robustly committed to the T lymphoid lineage as >90% were CD56^−^CD7^+^CD5^+^, and within this population, >95% were CD4^+^CD8αβ^+^ DP thymocytes (**Figures 2B, C** and **Supplementary Figure S6A**). As expected, those DP thymocytes were negative for the surface expression of the CD3/TCR complex (**Figure 2D**). The expression of mCherry and the CD38-CAR was efficiently induced on the surface after treatment with dox (**Figure 2E** and **Supplementary Figure S6B**). Interestingly, upon CAR expression, the DP thymocytes uniformly matured to single-positive CD8αβ^+^ CAR-iT cells (**Figure 2E** and **Supplementary Figure S6B**), probably receiving an activation/maturation signal due to the engagement of the CD38-CAR on CD38 expressed on the surface of the DP cells. Notably, CD38-mediated maturation resulted in approximately 30% survival of single-positive CD8αβ^+^ CAR-iT cells that had downregulated CD38 expression (**Supplementary Figures S6C, D**). DP thymocytes from TCRα^−/−^ indCD19CAR T-iPSC (clone 1.5–19 G8, **Table 1**) did not mature to CD8 SP iT cells upon the addition of dox, indicating that antigen encounter is required for efficient maturation. Indeed, CD8 SP CD19CAR iT cells could only be generated upon co-culture with irradiated CD19-expressing 3T3 cells (**Supplementary Figure S6E**). A CAR-mediated maturation is also supported by an observed reduction of CD8β expression (**Figure 2E**), which has been reported on primary T cells after stimulation (20) as well as CAR-iT cells (21). CD8αβ^+^ CAR-iT cells had an effector memory phenotype (CD45RA^−^CD62L^−^CCR7^−^) and expressed the costimulatory molecules CD27 and CD28 (**Supplementary Figure S6F**). We further assessed the potential of TCR^neg^CD8αβ^+^ CAR-iT cells for a specific CAR-mediated but not TCR-mediated response. After 48 h of dox treatment, CAR mRNA and protein surface expression returned to background after 7 days of expansion without dox (**Figure 2F** and **Supplementary Figures S6G, H** and **S7**). Only dox-treated TCR^neg^CD8αβ^+^indCD38CAR-iT cells, but not untreated TCR^neg^ iT cells, could proliferate in a standard mixed lymphocyte culture assay with an allogeneic feeder mix consisting of CD38^+^ Epstein–Barr virus-immortalized lymphoblastoid cell lines (EBV-LCLs) and peripheral blood mononuclear cells (PBMCs) from three donors (19) (**Figure 2G**), confirming the lack of alloreactivity of the *TCRα*^−/−^ indCAR-iT cells (**Figure 2G**). *TCRα*^−/−^ indCAR-iT cells with a CD38- or a CD19-CAR could expand 8- to 12-fold following weekly antigen stimulations (**Figure 2G** and **Supplementary Figures S6I, J**). These results demonstrate that timely expression of the CAR allows for efficient and robust generation of CD4^+^CD8αβ^+^ DP thymocytes that are able to mature and expand into functional non-alloreactive TCR^neg^CD8αβ^+^ indCAR-iT cells.

### TCR^neg^CD8αβ^+^ indCAR-iT cells display anti-tumor efficacy *in vitro* and *in vivo*

**Figure 3:**
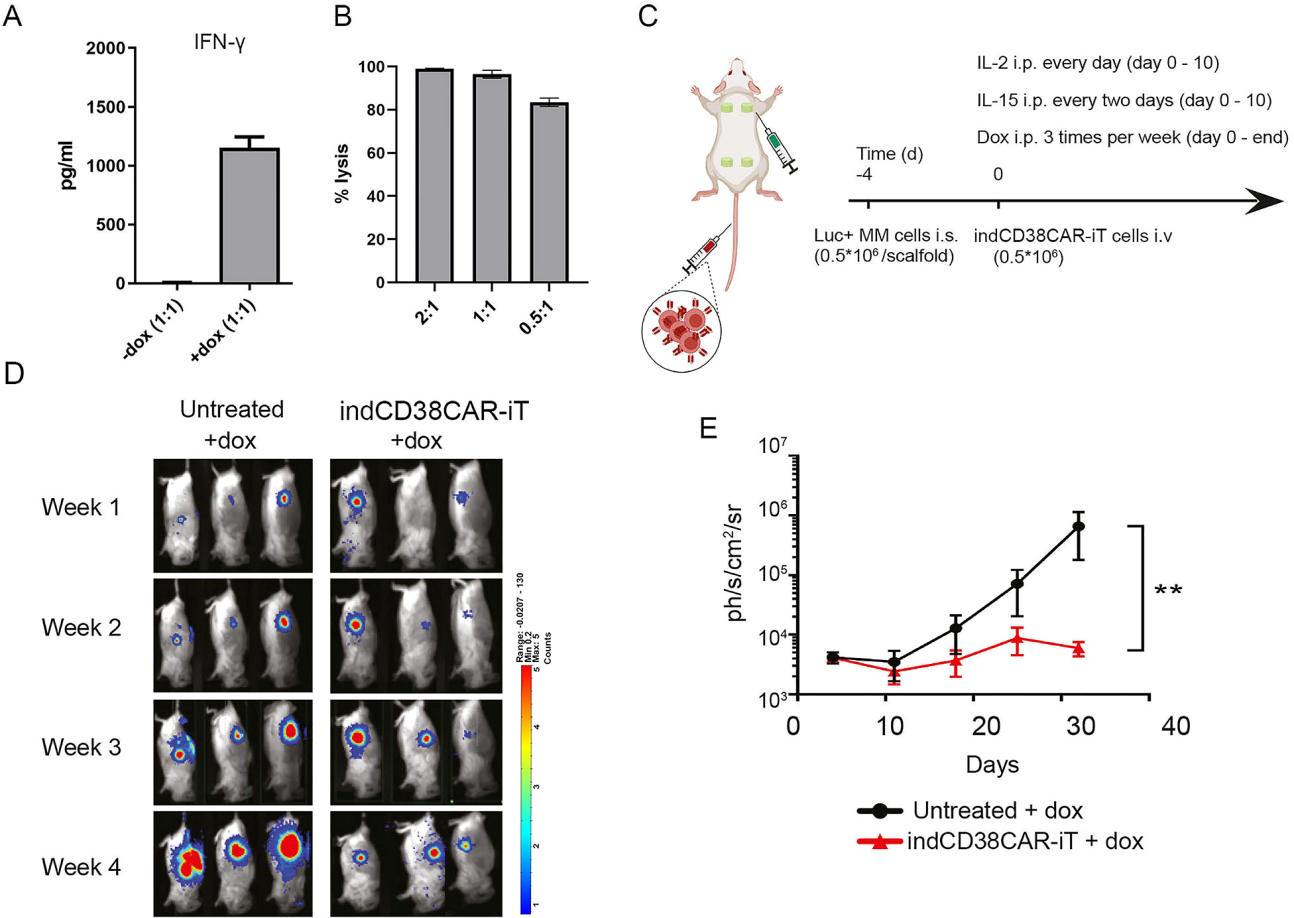
TCR^neg^CD8αβ^+^ indCD38CAR-iT cells exhibit anti-tumor activity. **(A)** Measurement of IFN-γ secretion by GFP9 CD8αβ^+^ CAR-iT cells after co-culture with CD38^+^ UM9 cells (E:T 1:1) with or without dox treatment (*n* = 2 technical replicates). Data are presented as mean ± SD. **(B)** Bioluminescence-based *in vitro* cytotoxicity assay of TCR^neg^CD8αβ^+^ indCD38CAR-iT cells against UM9 Luc-GFP cells for 24 h. Data are presented as mean ± SD. **(C)** Schematic representation of the xenograft multiple myeloma mouse model. UM9 Luc-GFP cells were injected intra-scaffold, followed 4 days after by intravenous injection of GFP9 TCR^neg^ CD8αβ^+^ indCD38CAR-iT cells + dox or dox only. IL-2, IL-15, and dox were injected i.p. as described. **(D)** Tumor burden was measured by BLI weekly. Images here shown with the pixel intensity represented in color. **(E)** Average tumor burden was quantified by BLI and is depicted as units per second per square centimeter per steradian (ph/s/cm^2^/sr) for the dox-only group (*n* = 4 mice) and the GFP9 TCR^neg^ CD8αβ^+^ indCD38CAR-iT cells + dox group (*n* = 3 mice). Data are presented as mean ± SEM. Statistical significance was calculated by Mann–Whitney test. **p < 0.01. Panel **(C)** was Created in BioRender. Themeli, M. (2026) https://BioRender.com/jjl5kt.

We further assessed the CAR-mediated anti-tumor potential of the TCR^neg^CD8αβ^+^ indCAR-iT cells. TCR^neg^CD8αβ^+^ indCD38CAR-iT cells were able to secrete IFN-γ upon co-culture with a CD38^+^ MM cell line, only upon dox treatment (**Figure 3A**). Also, expanded indCD38CAR-iT cells exerted tumor lysis against CD38^+^ MM cells (**Figure 3B**). In order to further interrogate the anti-tumor potency of CAR-iT cells *in vivo*, we used a scaffold-based, humanized bone marrow (BM), xenograft murine model of MM (22), where luciferase-expressing CD38^+^ UM9 cells were engrafted on the BM-like scaffold niches in the flanks of immunodeficient mice (**Figure 3C**). Administration of a single intravenous (i.v.) dose of TCR^neg^CD8αβ^+^ indCD38CAR-iT cells and intraperitoneal (i.p.) injection of dox resulted in a significant delay in MM tumor load compared to control mice receiving only dox (**Figures 3D, E**). Taken together, our data demonstrate that indCD38CAR-iT cells, generated from engineered T-iPSC, exhibit robust tumor lytic potential and can successfully control tumor growth *in vivo*.

### Generation of *TCRα*^−/−^*B2M*^−/−^HLA-E^+^ indCAR-T-iPSC

Studies have shown that silencing the expression of β2-microglobulin (β2M), which is a universal accessory protein across all HLA class I molecules, was able to safeguard PSCs from alloreactive CD8^+^ T cells (14, 23). Since abrogation of HLA class I expression would render PSCs vulnerable against NK cell-mediated rejection, additional overexpression of non-classical HLA molecules, such as HLA-E, has been shown to successfully protect PSCs against an NK-mediated attack (14, 16).

**Figure 4:**
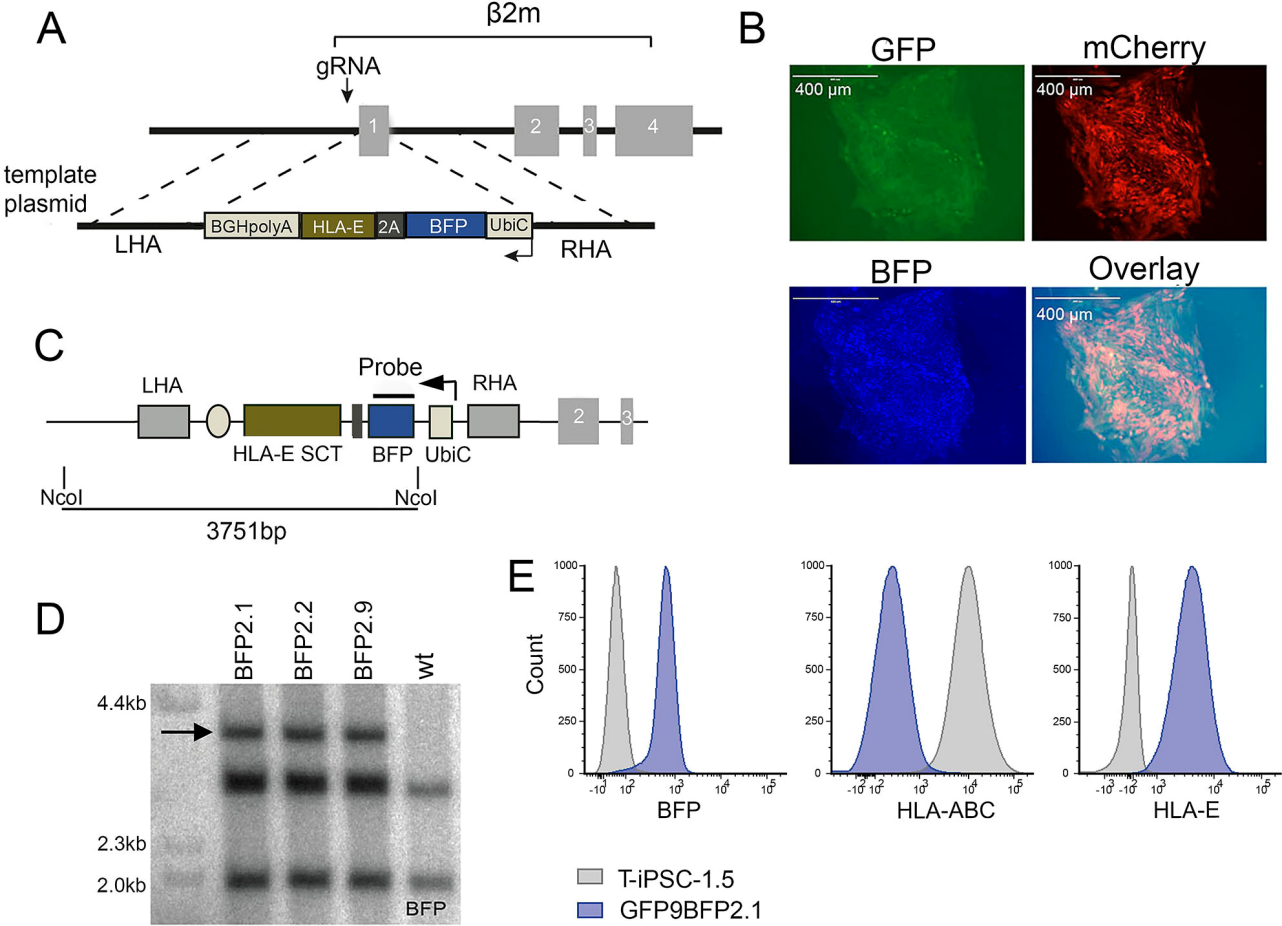
Establishment of *TCRα^−/−^B2M^−/−^*HLA-E^+^ indCAR-T-iPSC clones. **(A)** Schematic strategy for bi-allelic disruption of the *B2M* locus and insertion of a cassette for HLA-E SCT expression. Arrows show the position of the double-strand break at the beginning of exon 1. **(B)** Representative fluorescence microscopy images confirming the successful generation of TCRα^−/−^β2m^−/−^HLA-E^+^ indCAR-T-iPSC clones via the expression of GFP, mCherry, and BFP. Scale bar, 400 μm. **(C)** Schematic representation of Southern blot strategy to confirm the specific integration of the HLA-E SCT-BFP donor sequence. The area of interest is digested with NcoI and is targeted with a BFP-specific radiolabeled probe. **(D)** Southern blot analysis showing the successful insertion of HLA-E SCT on GFP9BFP T-iPSC clones by the presence of a band at 3.7 kb. **(E)** Expression of BFP, HLA-ABC, and HLA-E on GFP9BFP2.1 TCRα^−/−^β2m^−/−^HLA-E^+^ indCAR-T-iPSC compared to the WT.

Given those findings, we set out to establish T-iPSC lines that would incorporate the aforementioned B2M/HLA modifications towards the ultimate goal of producing hypoimmunogenic T-iPSC. To this end, we used our previously established GFP9 T-iPSC clone to further genetically edit its B2M locus. This time, we knocked-in in exon 1 of *B2M* the coding sequence of a previously described HLA-E SCT (18, 24) linked to a blue fluorescent protein (BFP) marker (**Figure 4A**). We isolated 10 GFP^+^BFP^+^ T-iPSC colonies (**Figure 4B**) and confirmed the successful integration of the BFP-HLA-E SCT transgene by PCR in 9/10 (**Supplementary Figures S8A**, **S9**), while in 5/10 colonies, the transgene was biallelic integrated (**Supplementary Figures S8A**, **S9**). Southern blot verified specific integration of the transgene into the *B2M* locus of the selected GFP^+^mCherry^+^BFP^+^ T-iPSC lines (**Figures 4C, D** and **Supplementary Figure S10**), which could still express mCherry after dox treatment (**Figure 4B**). We confirmed that GFP^+^BFP^+^ T-iPSC lacked expression of HLA class I molecules and successfully expressed HLA-E on their cell surface (**Figure 4E** and **Supplementary Figure S8B**). Although the parental GFP9 line (*TCRα*^−/−^) maintained a normal karyotype, the analysis of *TCRα*^−/−^*B2M*^−/−^HLA-E^+^ T-iPSCs (clone 1.5 GFP9BFP2.1, **Table 1**) showed the presence of trisomy 12 (**Supplementary Figures S8C**, **S5**). This karyotypic pattern was further corroborated in an additional independent experiment targeting the *B2M* locus in the parental unmodified T-iPSC line (clone 1.5 BFP2.1, **Table 1**), where abnormal karyotype involving Chr12 [47,XY, + 12,t(12;12)(q10;q10)] was again observed in 50% of the metaphases (**Supplementary Figure S8C**). These results indicate that the chromosomal instability is probably correlated with the *B2M* knockout process. Since Cas9/gRNA cannot theoretically cause chromosomal trisomy, a role of the absence of B2M protein can be assumed.

### Efficient generation of functional and hypoimmunogenicTCR^neg^β2M^neg^HLA-E^+^ indCAR-iT cells

**Figure 5:**
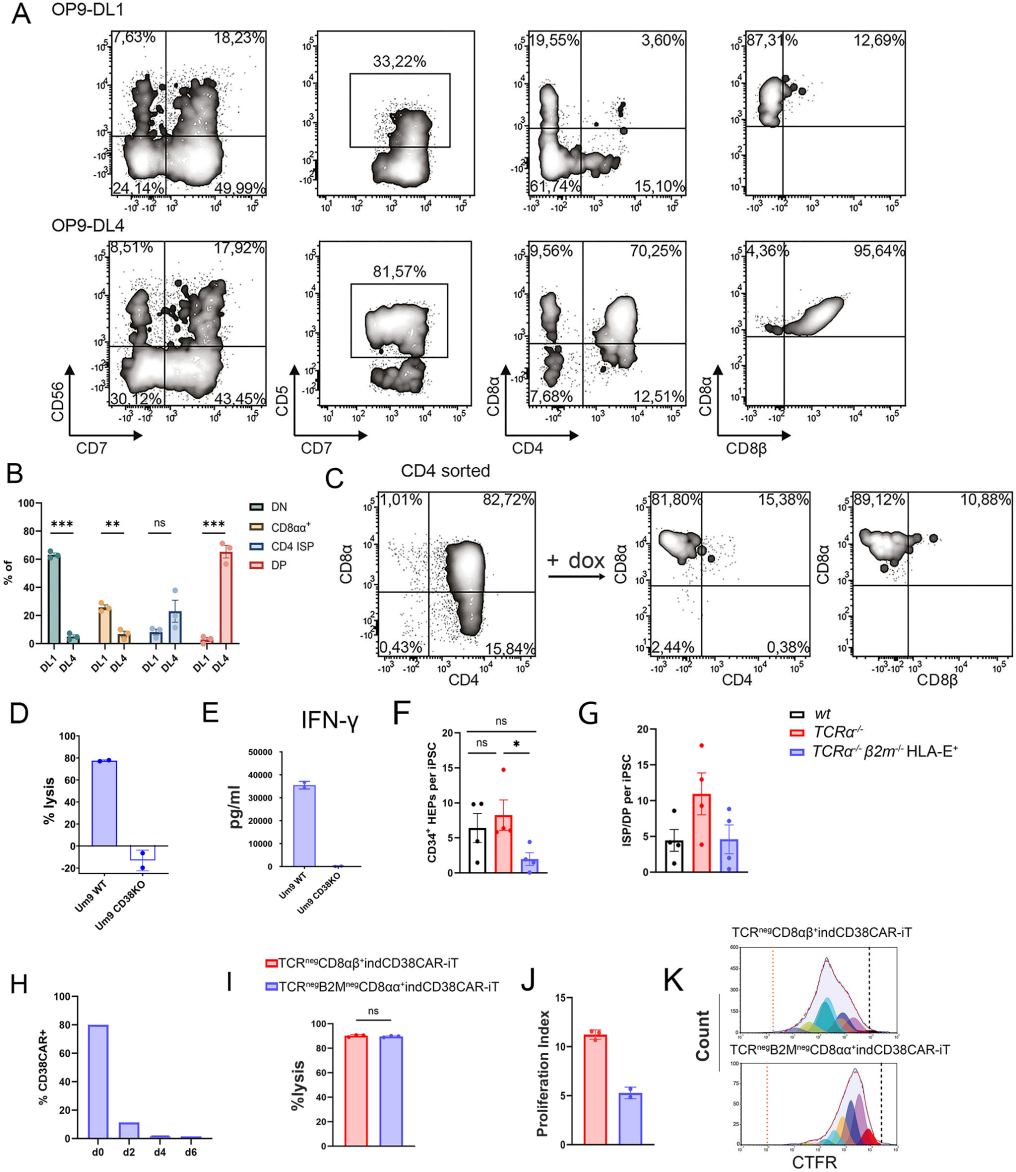
Efficient generation of functional TCR^neg^ B2M^neg^ HLA-E^+^ indCD38CAR-iT cells. **(A)** Representative flow cytometry plots showing expression of classical T-cell markers of TCR^neg^ B2M^neg^ HLA-E^+^ indCD38CAR-iT cells at Tdiff day 25 after co-culture with either OP9-DL1 or OP9-DL4 feeders. **(B)** Phenotype distribution of TCR^neg^ B2M^neg^ HLA-E^+^ indCD38CAR-iT without dox on day 25 of T-cell induction on OP9-DL1 or OP9-DL4 feeders (*n* = 3 independent differentiations). Data are presented as mean ± SEM. **(C)** Representative plot of purified TCR^neg^ B2M^neg^ HLA-E^+^ CD4 ISP and CD4^+^CD8αβ^+^ DP thymocytes at Tdiff day 25 after co-culture with OP9-DL4 cells. The addition of dox for 48 h results in maturation into TCR^neg^ B2M^neg^ HLA-E^+^ CD8αα^+^ indCD38CAR-iT cells. **(D)** TCR^neg^ B2M^neg^ HLA-E^+^ CD8αα^+^ indCD38CAR-iT cells display antigen-specific lysis. TCR^neg^ B2M^neg^ HLA-E^+^ CD8αα^+^ indCD38CAR-iT cells were co-cultured with irradiated EBV-LCLs for 1 week. Expanded cells were co-cultured with UM9 WT or CD38KO Luc-GFP cells at E:T 2:1 for 24 h and lysis was measured based on bioluminescence signal (*n* = 2, technical replicates). Data are displayed as mean ± SD. **(E)** Levels of secreted IFN-γ after co-culture of expanded TCR^neg^ B2M^neg^ HLA-E^+^ CD8αα^+^ indCD38CAR-iT cells with UM9 WT or CD38KO Luc-GFP cells at E:T 2:1 for 24 h (*n* = 2, technical replicates). Data are presented as mean ± SD. **(F)** Cell yield of CD34^+^ HEPs per T-iPSC of *wt* (*n* = 4 independent differentiations), *TCRα*^−/−^ (*n* = 4 independent differentiations), and *TCRα*^−/−^*B2M*^−/−^HLA-E^+^ T-iPSC (*n* = 4 independent differentiations). Data are presented as mean ± SEM. Statistical significance was calculated by unpaired student’s t-test or Mann–Whitney test. **(G)** Cell yield of ISP/DP cells per T-iPSC of *wt* (*n* = 4 independent differentiations), *TCRα*^−/−^ indCD38-CAR (*n* = 4 independent differentiations), and *TCRα*^−/−^*B2M*^−/−^HLA-E+ indCD38CAR-T-iPSC (*n* = 4 independent differentiations). **(H)** Surface CD38 CAR expression kinetics of TCR^neg^ B2M^neg^ HLA-E^+^ CD8αα^+^ indCD38CAR-iT cells upon dox withdrawal. **(I)** Bioluminescence-based *in vitro* cytotoxicity assay of TCR^neg^CD8αβ+ indCD38CAR-iT and TCR^neg^ B2M^neg^ HLA-E^+^ CD8αα^+^ indCD38CAR-iT cells against UM9 Luc-GFP cells (E:T 1:1) for 24 h (*n* = 3, technical replicates). Data are presented as mean ± SD. Statistical significance was calculated by unpaired student’s t-test. **(J)** Proliferation potential of TCR^neg^CD8αβ^+^ indCD38CAR-iT (*n* = 3 technical replicates) and TCR^neg^ B2M^neg^ HLA-E^+^ CD8αα^+^ indCD38CAR-iT cells (*n* = 2 technical replicates) upon co-culture with an allogeneic feeder mix. CAR-iT cells were stained with Cell Trace Far Red (CTFR) and proliferation capacity was evaluated by proliferation index. Data are presented as mean ± SD. **(K)** Generation peaks of TCR^neg^CD8αβ^+^ indCD38CAR-iT and TCR^neg^ B2M^neg^ HLA-E^+^ CD8αα^+^ indCD38CAR-iT as shown by CTFR staining during proliferation. *p < 0.05; ns, not significant.

After having established the *TCRα*^−/−^*B2M*^−/−^HLA-E^+^ T-iPSC clones, we further set out to differentiate them towards *TCRα*^−/−^*B2M*^−/−^HLA-E^+^ indCAR-iT cell derivatives. Interestingly, the *TCRα*^−/−^*B2M*^−/−^HLA-E^+^ T-iPSC, when differentiated on OP9-DL1 feeder cells, failed to generate CD4^+^CD8αβ^+^ DP cells, but only gave rise to CD56^−^CD7^+^CD5^low^ lymphoid cells (**Figure 5A**). The use of the DLL4 Notch-ligand can rescue the developmental skewing of T-iPSC caused by the premature expression of the TCR (13). We thus hypothesized that the use of OP9-DL4 may also improve the development of DP cells from *TCRα*^−/−^*B2M*^−/−^HLA-E^+^ T-iPSC. Indeed, when using OP9-DL4 feeder cells, we succeeded in generating CD7^+^CD5^+^ cells from *TCRα*^−/−^*B2M*^−/−^HLA-E^+^ T-iPSC, which were, in their majority, CD4^+^CD8αβ^+^ DP (**Figures 5A, B**). These data suggest that lack of B2M can influence the T lymphoid potential of T-iPSC and the use of DL4 can partially rescue the developmental block. We observed the same decrease of DP cell generation when starting from *TCRα^+/+^B2M*^−/−^ T-iPSC versus wild-type T-iPSC (**Supplementary Figure S11A**).

We further proceeded in investigating the functional potential of the TCR^neg^β2M^neg^HLA-E^+^ indCAR-iT cells. To exclude any contamination of innate T cells, we first performed purification of the total CD4^+^ population [including the DP and CD4 immature single-positive (ISP) cells]. The addition of dox induced, as expected, the maturation of the DP cells to CD8^+^ SP cells (**Figure 5C**). However, the expression of CD8β was decreased on the surface of the TCR^neg^β2M^neg^*^-^*HLA-E^+^ indCAR-iT cells after maturation. Nevertheless, the TCR^neg^β2M^neg^HLA-E^+^ indCD38CAR-iT cells were able to elicit specific lysis of the CD38^+^ UM9 MM cell line, while leaving intact the CD38-knockout derivative of the same cell line (UM9 CD38KO) (**Figure 5D**). This was further corroborated by analysis of cytokine secretion, where only co-culture with UM9 cells resulted in secretion of IFN-γ in contrast to co-culture with UM9 CD38KO cells (**Figure 5E**). Phenotypically, the cells displayed a terminally differentiated effector phenotype (CD45RA^+^CD62L^−^CCR7^−^) expressing the costimulatory receptor CD27 but not CD28 (**Supplementary Figure S11B**). Furthermore, *TCRα*^−/−^*B2M*^−/−^HLA-E^+^ T-iPSC yielded lower numbers of CD34^+^ hematopoietic progenitors (**Figure 5F**) and ISP/DP cells (**Figure 5G**) compared to both unmodified and *TCRα*^−/−^ T-iPSC. In line with the TCR^neg^ indCAR-iT cells, TCR^neg^β2M^neg^HLA-E^+^ indCAR-iT cells displayed downregulation of CAR expression within 1 week after dox withdrawal (**Figure 5H** and **Supplementary Figure S11C**). Although we did not see any difference on the cytotoxic potential of TCR^neg^B2M^neg^HLA-E^+^ indCD38CAR-iT cells compared to TCR^neg^ CD8αβ^+^ indCD38CAR-iT cells (**Figure 5I**), we did however observe that *B2M* knockout indCD38CAR-iT cells showed a lower proliferative potential (**Figures 5J, K**).

**Figure 6:**
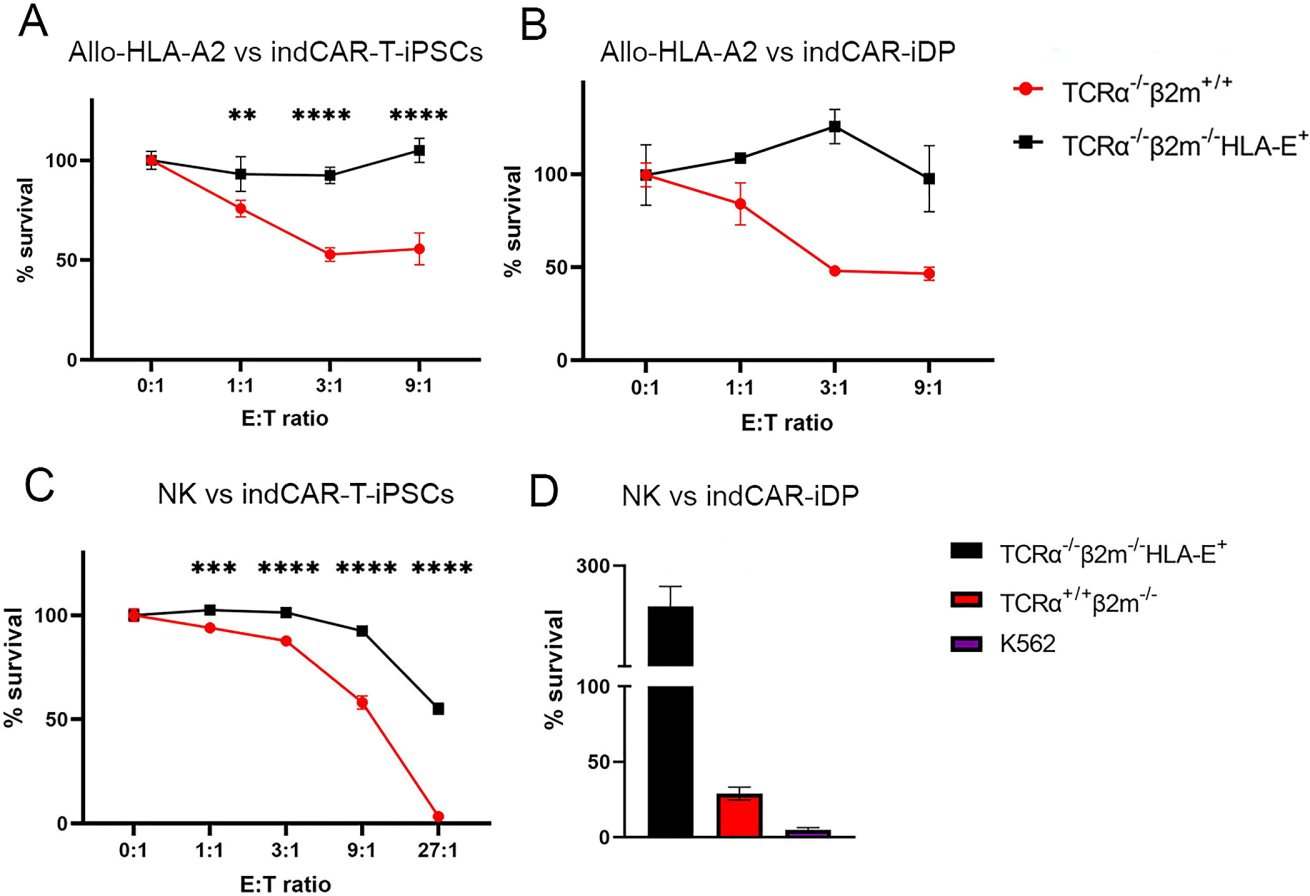
*TCRα*^−/−^*B2M*^−/−^HLA-E^+^ indCAR-T-iPSC and CD8αβ^+^ indCAR-iDP thymocytes are hypoimmunogenic. **(A, B)** Flow cytometry-based cytotoxicity assay of allo-HLA-A*02 T cells against *TCRα*^−/−^*B2M^+/+^* or *TCRα*^−/−^*B2M*^−/−^HLA-E^+^ indCAR-T-iPSC **(A)** and indCAR-iDP thymocytes **(B)** after co-culture for 24 h (*n* = 3, technical replicates; *n* = 2 technical replicates, respectively). **(C, D)** Flow cytometry-based cytotoxicity assay of NKG2A^+^ NK cells against TCRα^−/−^β2m^+/+^ or TCRα^−/−^β2m^−/−^HLA-E^+^ indCAR-T-iPSC **(C)** and indCAR-iDP thymocytes **(D)** after co-culture for 24 h; *n* = 3 technical replicates and *n* = 2 technical replicates, respectively. Survival percentage is calculated as the fold change in the cell numbers of the treated to the untreated conditions. Data are presented as mean ± SD. Statistical significance was calculated by 2-way ANOVA (A,C). **p < 0.01, ***p < 0.001, ****p < 0.0001.

Finally, we evaluated whether the engineered *TCRα*^−/−^*B2M*^−/−^HLA-E^+^ T-iPSC and indCAR-iT cells could trigger an immune response from allogeneic T cells and NK cells. Since the parental T-iPSC clone carries the HLA-A*02^+^ allele, we assessed the lysis of engineered T-iPSC clones expressing firefly luciferase and their derivative iDP cells by an alloreactive anti-HLA-A*02 T cell clone (allo-HLA-A*02). Immunogenicity at the iDP cell level was performed without dox treatment in order to avoid CAR-mediated interactions with the CD38 molecule expressed on the effector cells in this assay. Although allo-HLA-A*02 T cells effectively lysed the parental GFP9 T-iPSC and their derivative *TCRα*^−/−^ indCD38CAR-iT cells, abrogation of B2M and lack of HLA class I expression was able to confer protection in the *TCRα*^−/−^*B2M*^−/−^HLA-E^+^ T-iPSC clone (**Figure 6A**) as well as the TCR^neg^β2M^neg^HLA-E^+^ indCAR-iT cells (**Figure 6B**). Furthermore, as expected, the lack of HLA class I expression resulted in the lysis of *TCRα*^−/−^*B2M*^−/−^ T-iPSC (**Figure 6C**) and *TCRα*^−/−^*B2M*^−/−^ iDP cells from primary NKG2A^+^ NK cells (**Figure 6D**). The overexpression of HLA-E SCT, which was inserted in the *β2m* locus, was able to successfully compensate for the lack of HLA class I molecules and safeguard the recognition of *TCRα*^−/−^*B2M*^−/−^HLA-E^+^ T-iPSC and iDP cells from NKG2A^+^ NK cells (**Figures 6C, D**).

## Discussion

The use of allogeneic donor-derived T cells has been pursued as an alternative resource of therapeutic T cells in order to surpass the shortcomings of current autologous CAR-T cell manufacturing methods. An optimal allogeneic therapeutic T cell, however, would need to possess specific features, which require substantial genome engineering. Unfortunately, genetic engineering of primary T cells in a multiplex manner is very challenging, as it currently results in (a) reduced production yield; (b) genotoxicity due to undesired off-target effects and gene translocations; (c) an exhausted T-cell phenotype due to the requirement of extended *ex vivo* expansion; and (d) high production costs and effort for safety release testing (25). The potential of iPSC for unlimited culture and genetic editing makes them an attractive platform to produce multi-engineered, selected, and carefully screened clonal cell banks that can be used to generate homogenous T-cell products with desired therapeutic properties (9). We, here, show an efficient strategy to genetically engineer iPSC by utilizing well-characterized loci to knock-in desirable transgenes and robustly generate functional CD8αβ CAR-T cells, resistant to CD8^+^ T and NK cell-mediated rejection. Our data provide insights for optimal CAR expression strategies and underscore the importance of evaluating the impact of every engineering step to the functional quality of CAR-iT cells.

Previous studies have reported strategies to confer human embryonic stem cells (ESCs) or iPSC hypoimmunogenic and resistant to T- and NK-cell rejection including the elimination of HLA class I and class II molecule expression, the introduction of the non-classical HLA-E molecule, and the knockout of NK-cell activating ligands (CD155, B7-H3) (14–17, 26). In the majority of these studies, the engineered iPSC lines were made by numerous sequential genetic editing processes along with additional steps of retro/lentiviral delivery of transgenes (e.g., HLA-E and CAR), even at the iT stage. However, the accommodation of as many optimized immunotherapeutic properties as possible in the iPSC-derived CAR-T cells requires the facilitation of engineering processes.

Here, we propose that all required features of CAR-iT cells can be engineered at the iPSC level. Most importantly, the loci of the genes required to be knocked out can serve as insertion sites for the overexpression of transgenes whose expression is desired, reducing in this way the required steps of genetic engineering. Using CRISPR/Cas9, we knocked out the *TRAC* and *B2M* loci and utilized the edited alleles to insert the expression cassettes coding for a doxycycline-inducible CD38CAR and the HLA-E SCT. In our study, the two alleles of the *TRAC* locus were successfully simultaneously modified with 2 different gene expression cassettes by homologous recombination. The sequence of the tetracycline-controlled transactivator rtTA driven by the Ubic promoter was inserted in one allele while the coding sequences of the TRE and the CD38CAR were inserted in the other allele. In a separate editing step, the HLA class I expression was silenced by disrupting the *B2M* locus and the immune modulatory HLA-E molecule, which has been used in pre-clinical studies and clinical trials, in order to inhibit NK-cell-mediated rejection (16, 27). Thus, theoretically, when knocking out 2 genes, one has the opportunity to insert up to 4 different gene-expression cassettes. In our study, we limited the edited loci to 2, the *TRAC* and the *B2M*. However, the rationale of our study could be extended to other loci, which can be edited in order to further ensure the lack of immunogenicity and improve the efficacy of CAR-iT cells (HLA class I or II genes, *CIITA*, *CD155*, *B7-H3*, *Pdcd1, CD52, CD38*, etc.) (14–17, 28, 29). Moreover, although we used T cell-derived iPSC in this study, this strategy could be theoretically applicable to iPSC of other origins.

Previous work on T-iPSC that are engineered to constitutively express a CAR has shown that these cells give rise mainly to CD8αα^+^ CAR-T cells with an expression profile and function similar to innate T cells, which lack some key features for therapeutic potency, such as the long-term memory and *in vivo* persistence (10, 13). Premature expression and signaling of an endogenous or transgenic TCRαβ (30, 31) as well as CAR-mediated tonic signaling can cause a block in T-cell development (32). Differentiation using an artificial thymic organoid system allowed the development of mature CD8αβ^+^ CD19CAR-iT cells from T-iPSC constitutively expressing a CD19CAR, suggesting that a 3D thymic-like structure might be important for T-cell development (14). Reducing the intensity of CAR-mediated tonic signaling by using a CD19CAR bearing mutated ITAMs and timing the CAR expression to a later time point during the differentiation process results in a partial rescue of β-selection and the emergence of CD4^+^CD8αβ^+^ DP iT cells (13). We, here, show that allowing CAR expression only at the DP stage of development, when physiologically the full TCRαβ complex is expected to be expressed, would allow the unobstructed and robust development of CD4^+^CD8αβ^+^ DP iT cells. Indeed, T-iPSCs engineered with the dox-inducible CD38CAR were efficiently differentiated to CD4^+^CD8αβ^+^ DP cells and upon dox treatment to CD8αβ^+^ CAR-iT cells (>95% efficiency). Previously, the use of a single lentiviral vector for the dox-inducible control of CAR expression allowed some development of DP iT cells up to 60% (21). We believe that, in our system, the precise editing and insertion of one transgene copy in the T-iPSC results in better control of CAR expression without leakiness. Of course our method is limited by the use of the non-eukaryotic tetracycline response transactivator, which complicates a clinical application. Nevertheless, our study showcases that robust production of mature CD8αβ^+^ CAR-iT cells from engineered T-iPSC is possible by using measures that efficiently withhold the expression of the CAR-transgene.

In contrast to the unimpeded emergence of CD8αβ^+^ CAR-iT cells from *TCRα*^−/−^ indCAR-T-iPSC, we found that knockout of *B2M* affected genomic stability and the efficiency of T lymphoid development. We observed karyotypic abnormalities in *B2M*^−/−^ T-iPSC, specifically the emergence of trisomy 12. Notably, similar chromosomal instability was detected in cells subjected to different independent rounds of *B2M* CRISPR knockout. Since theoretically trisomy cannot be a result of CRISPR off-target activity (33), we assumed a role of *B2M*^−/−^ in the propagation of genomic unstable clones. Indeed, a complete lack of β2M expression has been previously reported to slow the growth of normal, euploid ESCs (16). Moreover, trisomy 12 is a common aneuploidy described in human ESC and iPSC, possibly related to specific culture conditions (34). Overall, our findings and those of others suggest that *B2M*-edited iPSC should be carefully and regularly screened for genomic instability. Additionally, we found that *B2M*^−/−^ T-iPSC differentiated less efficiently to CD34^+^ and DP iT cells. There is limited knowledge on a potential role of β2M or HLA-class I expression in T-cell development before the stage of positive and negative selection. According to a recent report, the addition of human recombinant β2M increased thymocyte cellularity in a thymic epithelial cells/thymocyte culture system (35). Wang et al. have shown the development of CD8αβ^+^ iT cells from chromosomally stable *B2M*^−/−^ T-iPSC (14). Thus, although there are indications that β2M may have less studied, non-obvious functions, we cannot exclude that the negative impact on genomic stability and T-cell development may be connected and related to the iPSC culture conditions (use of MEF feeders) or even the iPSC donor source. Nevertheless, the emergence of β2M^neg^CD4^+^CD8αβ^+^ DP cells was partially restored when using OP9 bearing DL-4, a ligand with higher affinity for NOTCH1.

It is critical to assess the effects of any genomic editing on the functionality of the CAR-iT cells and confirm that the desired features are present without compromising the anti-tumor efficacy. Indeed, the lack of TCR rendered the CAR-iT cells non-alloreactive against a mix of allogeneic PBMCs, the lack of HLA class I expression protected them from lysis by anti-HLA-A*02 CD8^+^ T cells, while the overexpression of the HLA-E SCT made them immune to rejection from NKG2A^+^ NK cells. Importantly, knockout of the *TRAC* did not affect the anti-tumor functionality of TCR^neg^ CD8αβ^+^ indCD38CAR-iT cells, which responded to CD38 engagement *in vitro* by proliferating, secreting IFN-γ, and eliciting tumor lysis and successfully controlled tumor growth in a xenograft murine model for MM. Besides the reduced efficiency of *TCRα*^−/−^*B2M*^−/−^HLA-E^+^ T-iPSCs to differentiate them from TCR^neg^β2M^neg^CD8αβ^+^ DP cells, induction of the CD38CAR resulted in the emergence of TCR^neg^β2M^neg^CD8αα^+^ iT cells. Nevertheless, the TCR^neg^β2M^neg^CD8αα^+^ indCAR-iT cells did not show innate-like, non-antigen-dependent cytokine secretion or tumor lysis, which has been previously described for iPSC-derived CD8αα^+^ iT cells (36) and *B2M* knockout did not impair their antigen-dependent cytotoxic capacity. We did, though, observe that TCR^neg^β2M^neg^CD8αα^+^ indCAR-iT cells had significantly reduced proliferation upon antigen engagement compared to TCR^neg^CD8αβ^+^ indCAR-iT cells. Our data indicate a developmental and functional defect of the TCR^neg^β2M^neg^CD8αα^+^ iT cells. However, since we did not succeed in generating a chromosomally stable *B2M*^−/−^ T-iPSC line (**Supplementary Figure S8C**), we cannot rule out a potential influence of the abnormal karyotype alone or in combination with B2M absence. Nevertheless, previous studies have also investigated the effect of B2M silencing on T-iPSCs or ESCs, which maintained normal karyotype. Despite the fact that no significant impact in the overall function of TCR- or CAR-iT cells was reported, careful evaluation of individual experiments showed cases of lower cell yield as well as slight reduction of lytic and proliferative performance *in vitro* (14, 26).

In summary, we have succeeded in exploiting well-characterized genomic loci to engineer T-iPSC and generate mature, functional TCR^neg^β2M^neg^ indCAR-iT cells. The inducible CAR expression allows for an unpreceded robust efficiency of production of CD4^+^CD8αβ^+^ DP and CD8αβ^+^ CAR-iT cells. Our data suggest that the choice of target loci should be based on careful assessment of the impact of genetic modifications on the genomic stability and the developmental potential of iPSC as well as on the overall functional potential of the produced effector CAR-iT cells. The facilitation of multi-engineering at the iPSC level along with the improved production efficiency can further enable the use of iPSC as a powerful source of therapeutic T cells.

## Materials and methods

### Cell lines

The human multiple myeloma (MM) cell line UM9 (obtained from UMC Utrecht) [unmodified, lentivirally transduced to express luciferase (Luc-GFP) or with silenced CD38 expression (CD38 KO)] was cultured in Roswell Park Memorial Institute (RPMI)-1640 medium (Thermo Fisher Scientific, Cat#21875091) along with 10% HyClone Fetal Clone I (Fisher Scientific, Cat#SH30080.03), 100 U/mL penicillin, and 100 µg/mL streptomycin (Thermo Fisher Scientific, Cat#15140122). EBV-LCLs (obtained from UMC Utrecht) were cultured in RPMI-1640 medium (Thermo Fisher Scientific) supplemented with 10% fetal bovine serum [FBS (Sigma-Aldrich), Cat#F7524], 100 U/mL penicillin, and 100 µg/mL streptomycin. CF-1 mitomycin C-treated mouse embryonic feeder cells [MEFs (Tebu Bio), Cat#MEF-MITC] were maintained in Dulbecco’s Modified Eagle Medium (DMEM) high-glucose Glutamax (Thermo Fisher Scientific, Cat#10569010) supplemented with 10% FBS, 100 U/mL penicillin, and 100 µg/mL streptomycin. The OP9-DL1 and OP9-DL4 cell lines (kind gift from Dr. Michel Sadelain, Memorial Sloan Kettering Cancer Center) were cultured in minimum essential medium alpha (MEMα) with no nucleosides (Fisher Scientific, Cat#12000014), 0.2% sodium bicarbonate (Thermo Fisher Scientific, Cat#25080094), 2 mM L-glutamine (Thermo Fisher Scientific, Cat#25030024), and 20% FBS Hyclone Characterized (Fisher Scientific, Cat#SH30071.03). The Allo-HLA-A*02 T cell clone (obtained from UMC Utrecht) was cultured with Iscove’s Modified Dulbecco’s Medium [IMDM (Thermo Fisher Scientific), Cat#21980032] supplemented with 10% Human Heat Inactivated AB Serum (Sigma-Aldrich, Cat#H3667), 100 U/mL penicillin, 100 µg/mL streptomycin, and 25 U/mL recombinant human (rh) IL-2 [(Proleukin) Novartis, Cat#4764032NL]. They were weekly stimulated by co-culturing 200,000 Allo-HLA-A*02 T cells with 1 × 10^6^ irradiated feeder-PBMCs from three donors (25 Gy) and 1 × 10^5^ EBV-LCLs (50 Gy) in medium supplemented with 25 U/mL rhIL-2 and 1 µg/mL Phytohemagglutinin-Ligand (PHA-L; Sigma-Aldrich, Cat#L4144). The fibroblast cell line NIH/3T3 (ATCC CRL-1658), lentivirally transduced to overexpress CD19, was cultured on DMEM high-glucose Glutamax supplemented with 10% FBS, 100 U/mL penicillin, and 100 µg/mL streptomycin. All cell lines were incubated at 37 °C with 5% CO_2_ and were regularly checked with Short Tandem Repeat (STR) analysis and tested for mycoplasma.

### Primary human cells

PBMCs were isolated from fresh healthy donor buffy coats, purchased by Sanquin Blood bank after informed consent. NKG2A^+^ NK cells were sorted via FACS as CD3^−^CD56^+^NKG2A^+^ in a BD FACSAria™ III Cell Sorter. Isolated cells were then expanded by being co-cultured with irradiated (100 Gy) K562 cells (E:T 3:1) in RPMI-1640, 10% Human Heat-Inactivated AB Serum, 100 U/mL penicillin and 100 µg/mL streptomycin supplemented with 100 U/mL rhIL-2, 10 ng/mL rhIL-15 (Peprotech, Cat#200-15), and 10 ng/mL rhIL-21 (R&D Systems, Cat#8879-IL-010). For some experiments, NKG2A^+^ NK cells were kindly offered by the group of Dr. Lotte Wieten (Maastricht University Medical Center).

### Maintenance of T-iPSC

The original T-iPSC clone 1.5 was a kind gift of Dr. Michel Sadelain (Memorial Sloan Kettering Cancer Center). They were cultured on CF-1 mitomycin C-treated MEF cells at approximately 7,500 MEF cells/cm^2^ in iPSC medium consisting of DMEM/F12 (Thermo Fisher Scientific, Cat#11330-057) along with 20% KnockOut Serum Replacement (KSR, Cat#10828-028), 2 mM L-glutamine, 1% nonessential amino acids (NEAA, Cat#11140050), and 55 μM 2-mercaptoethanol (Cat#21985023) (all Thermo Fisher Scientific). The medium was enriched with 10 ng/mL human basic fibroblast growth factor (hbFGF) Peprotech], Cat#100-18C-B). T-iPSCs before nucleofection or viral transduction were cultured on plates coated with Cultrex Basement Membrane Extract [(BME), R&D Systems, Cat#3434-010-02] in mTeSR™ Plus medium (STEMCELL Technologies, Cat#100-0276). For induction of T-cell differentiation based on a monolayer system, T-iPSCs were cultured on Matrigel^®^ Matrix (Corning, Cat#354234) at 1:40 dilution. Medium was refreshed every day and passaging of cells was performed upon treatment with 1 U/mL Dispase (STEMCELL Technologies, Cat#07923) at a ratio of 1:3, every 4–5 days. Freezing of iPSCs was performed in medium consisting of 60% KSR, 20% iPSC medium and 10% dimethyl sulfoxide [DMSO (Sigma-Aldrich), D8418-250ml]. Cryovials (Greiner BIO-ONE, Cat#123263) were placed on a Mr Frosty device (Thermo Fisher Scientific, Cat#5100-0001) and stored at −80°C for 24 h, before being transferred to liquid nitrogen barrels for long-term storage. Frozen cells were thawed on DMEM F/12 medium at 1:10 ratio, centrifuged at 300*g* and seeded on MEFs with iPSC medium. Characterization and cryopreserving of each clone were performed on passage number described in **Table 1**. In principle, each iPSC clone was kept in culture for approximately 15 passages. Pluripotency was tested by flow cytometry upon clone generation, measuring the expression of markers TRA-1-60, TRA-1-81, SSEA-3, and SSEA-4 (data not shown). Mycoplasma testing was performed every 6 months by Eurofins Nederland.

### Mouse strains and animal care

RAG-2^−/−^γc^−/−^ female mice were bred and maintained at the Amsterdam Animal Research Center (Universitair Proefdiercentrum, AARC-UPC) under proper environmental conditions with free access to water and food. The animal experiments were performed under the approval of the central authority for scientific procedures on animals (CCD) (protocol number AVD114002015345). In strict accordance with the Dutch Animal Experimentation Act, animal welfare was monitored, and euthanasia was induced. Mice (with a minimum of *n* = 4 per group) were randomly assigned to treatment groups and the anti-tumor efficacy was analyzed.

### Generation of genome-edited T-iPSC clones

For the generation of the TCR^−/−^indCAR clones, CRISPR/Cas9-directed homologous recombination was used. The transgene donor template sequences were integrated in pBluescript (pBS) vector plasmids containing right and left homology arms flanking the starting codon of exon 1 of the *TRAC* locus (kind gift of Dr. Sadelain, MSKCC) (**Figure 1A**). One donor vector contained the sequence encoding the rtTA and a GFP reporter gene under the control of the Ubic promoter (pBS-TRAC-Ubic-GFPrtTA). The second template contained the sequence of a previously described CD38-CAR (CD38-28ζ) (19) or CD1928ζ-CAR (10), linked to the mCherry reporter gene, under the transcriptional control of a tetracycline response element (TRE). Both cassettes were inserted in reverse orientation. For targeted integration in the *B2M* locus, the homology arms of the template vector were replaced with sequences flanking the starting codon of *B2M.* The insert sequence contained the BFP reporter gene and the HLA-E SCT as described in Crew et al. (2005) (kind gift of Dr. Seebach, University Hospital of Zürich), driven by the Ubic promoter.

Targeted delivery of the above sequences into the T-iPSC was mediated via CRISPR/Cas9 genomic editing. Briefly, undifferentiated T-iPSC colonies cultured on Cultrex BME-coated plates were dissociated into single cells with Accutase (Thermo Fisher Scientific, Cat#A1110501). For each condition, 4 × 10^6^ cells were used, which were resuspended in 100 µL of Amaxa Cell Line Nucleofector Kit V solution (Lonza, Cat#VCA-1003). From each template plasmid, 2.6 µg was added along with 2.6 µg of the pX330-U6-Chimeric_BB-CBh-hSpCas9 vector (Addgene), which carried the spCas9 gene and a gRNA specific for the *TRAC* or the *B2M* locus. Electroporation took place in an Amaxa Nucleofector II device (Lonza) using program B-025, and after nucleofection, cells were transferred on MEF-plated dishes along with 10 µM Y-27632 (Selleckchem, Cat#S1049). Single cell-derived colonies were picked according to reporter gene expression (GFP, mCherry and BFP) using fluorescent microscopy (EVOS FL fluorescence microscope, Life Technologies) or flow cytometry.

Molecular verification of successful genomic editing was performed with PCR using the Phusion Hot Start II High-Fidelity Mastermix (Thermo Fisher Scientific) and specific primer pairs (sequences described in **Table 2**). Briefly, for detecting the template insertion in both alleles of the *TRAC* locus, primers were designed binding or flanking the left and right homology arms (TRAC-1 and TRAC-2). In addition, the insertion of both separate template sequences was confirmed by PCR with primers specific for amplifying GFP and mCherry coding sequences (GFP-1 and -2, mCherry-1 and -2). Similarly, to confirm the insertion of BFP and HLA-E genes in the *B2M* locus, we performed a PCR with primers amplifying a targeted version of the locus (primers β2m-1 and β2m-2) and a second PCR with primers specific for the non-modified locus (primers β2m-3 and β2m-4) (see **Table 2**).

Karyotypic analysis of the edited clones was performed by the Department Human Genetics at VU Medical Center. Verification of editing was performed upon sufficient expansion of each generated line, which took place approximately one to two passages after each round of genome editing.

### RT-PCR for CAR mRNA detection

RNA was isolated from iT cells on different phases of dox treatment by using TRIzol™ Reagent (Thermo Fisher Scientific, Cat#15596026) following the manufacturer’s guidelines. cDNA was subsequently synthesized by using the SuperScript™ IV First-Strand Synthesis System (Thermo Fisher Scientific, Cat#8091050). For RT-PCR, the Phusion Hot Start II High-Fidelity PCR Master Mix (Thermo Fisher Scientific, Cat#F565S) was used for amplification by employing CD38 CAR-E2A-mCherry cDNA primers 1 and 2 (see **Table 2**).

### Generation of UM9-CD38KO cells

UM9 cells were transfected with the pX330-U6-Chimeric_BB-CBh-hSpCas9 vector containing a gRNA for CD38 (see **Table 2**). After expansion, UM9-CD38KO cells were selected after sorting based on CD38 negativity.

### Southern blot

Genomic DNA of T-iPSC clones was obtained by phenol/chloroform extraction. Southern blot analysis was carried out with 10 µg of genomic DNA digested with the appropriate restriction enzymes (BamHI or NcoI). The probes corresponding to GFP (299-bp fragment), mCherry (300-bp fragment), and BFP (302-bp fragment) were generated by PCR and labeled with 32P-dCTP using the Random Primers DNA Labeling System (Invitrogen).

### Western blot

T-iPSC colonies treated with or without dox were lysed for 45 min in radioimmunoprecipitation assay (RIPA) lysis buffer (50 mM Tris, pH 7.4, 150 mM NaCl, 1% NP40, 0.5% sodium deoxycholate, and 0.1% SDS) supplemented with cOmplete™, Mini Protease Inhibitor Cocktail (Roche, Cat#11836153001) to allow protein extraction. Protein amount was quantified by using a bovine serum albumin (BSA) standard curve. For immunoblotting, 10 µg of protein lysate was loaded on 4%–20% precast gels (Bio-Rad, Cat#4561095) and proteins were fractioned by sodium dodecyl sulfate polyacrylamide gel electrophoresis (SDS-PAGE). Gel was then transferred to polyvinylidene fluoride (PVDF) membranes (Millipore, Cat#IPFL00010). Blocking was performed by using 5% w/v non-fat dried milk powder (Nutricia, Cat#8712400117654) for at least 30 min in PBS/0.1% Tween (PBS/T). For CAR identification, membranes were incubated overnight with an anti-CD247 monoclonal antibody (1D4) 1:250, purchased from BD Biosciences, at 4°C. After washing with PBS/T, membranes were incubated with horseradish peroxidase-conjugated rabbit anti-mouse secondary antibody, 1:2,000 purchased from Cell Signaling Technology, for 1 h at room temperature. Actin detection was used as positive control by staining with a mouse anti-human actin antibody (C4), 1:5,000 purchased from Sigma-Aldrich. Results were visualized by digital fluorescence in Odyssey machine (Licor).

### Differentiation of T-iPSC into CAR-iT cells

T-iPSCs were differentiated into T lineage cells using a previously described protocol with modifications. In brief, undifferentiated T-iPSC colonies were treated with 1 U/mL Dispase and transferred into ultra-low attachment plates to allow embryoid body (EB) formation. EBs were cultured in StemPro-34 medium (Thermo Fisher Scientific, Cat#10639-011) supplemented with 2 mM L-glutamine, 1% NEAA, 55 μM 2-mercaptoethanol, 100 U/mL penicillin, 100 µg/mL streptomycin, and 50 mg/mL L-ascorbic acid (Thermo Fisher Scientific, Cat#A4544). EB formation was facilitated by overnight incubation along with 30 ng/mL rhBMP-4 (R&D Systems, Cat#314-BP-010/CF). Medium was refreshed the next day and EBs were then cultured with 30 ng/mL rhBMP4 and 5 ng/mL hbFGF until day 3 to promote mesoderm induction. Afterwards, hematopoietic specification was achieved by the addition of 20 ng/mL rhVEGF (Peprotech, Cat#100-20-B) and 5 ng/mL hbFGF as well as a cocktail of hematopoietic cytokines [100 ng/mL rhSCF (Cat#300-07_100μg), 20 ng/mL rhFlt3L (Cat#300-19B), and 20 ng/mL rhIL-3 (Cat#200-03B) (all Peprotech)]. Medium was refreshed every 2 days until day 9 (hbFGF was added until day 7), where EBs were dissociated by treatment with Accutase and mechanical disruption to allow egression of CD34^+^ cells.

For experiments regarding cell yield, a previously described monolayer protocol with slight modifications was used (37, 38). Briefly, on day 3, T-iPSC colonies were dissociated into single cells via treatment with Accutase and seeded onto Matrigel-coated plates (1:40 dilution) at a concentration of 4.2 × 10^4^ cells/cm^2^ cultured in mTeSR™ Plus medium supplemented with 10µM Y-27632. After 24 h, Y-27632 compound was removed and T-iPSCs were cultured in mTeSR™ Plus medium for an additional 2 days. On day 0, medium was changed into HEP medium consisting of RPMI-1640 medium without glutamine (Cat#31870074), 2% B-27™ (Cat#17504044), 2 mM GlutaMAX™ (Cat#35050061) (all from Gibco), and 60 μg/mL L-ascorbic acid (Sigma-Aldrich), supplemented with 6 μM GSK3 inhibitor CHIR990921 (Merck, Cat#S2924). On day 2, medium was changed into HEP medium along with 50 ng/mL rhVEGF and 10 ng/mL hbFGF. The following day (day 3), cells were replated onto Matrigel-coated plates at a concentration of 5.2 × 10^4^ cells/cm^2^ cultured with HEP medium along with 50 ng/mL VEGF and 10 ng/mL bFGF and 10 µM SB431542 (Merck Millipore, Cat#616461). Medium was refreshed on day 4, and on day 5, CD34^+^ hematoendothelial progenitors (HEPs) were cultured on OP9-DL4 feeder layers as described below.

T lineage commitment was induced by culturing generated hematopoietic progenitors into OP9-DL1 or OP9-DL4 monolayers in a T-cell differentiation medium consisting of OP9 medium along with 1% NEAA, 55 μM 2-mercaptoethanol, 100 U/mL penicillin, 100 µg/mL streptomycin, and 50 mg/mL L-ascorbic acid. For efficient T-cell generation, T differentiation medium was enriched with 10 ng/mL rhSCF, 5 ng/mL rhIL-7, and 10 ng/mL rhFlt3L (all Peprotech). Medium was refreshed every 2 days and differentiating cells were transferred into a new monolayer every 5 days until day 25, where the presence of CD4^+^CD8αβ^+^ DP cells was observed. OP9-DL1 or OP9-DL4 cells were seeded a day prior to the cell transfer at a concentration of 2.1 × 10^4^ cells/cm^2^ in OP9 cell culture medium. For further maturation into CD8^+^ SP T cells, CD4^+^CD8αβ^+^ DP thymocytes were seeded on OP9-DL1 or OP9-DL4 feeder layers in T-cell differentiation medium supplemented with rhIL-7, rhFlt3L, and rhSCF along with 1 µg/mL dox (Clontech Laboratories, Cat#631311). After 48 h, dox was washed out and cells were cultured in RPMI-1640 supplemented with 10% FBS, 100 U/mL penicillin, and 100 µg/mL streptomycin supplemented with 10 ng/mL IL-7, until used for downstream assays.

### *In vitro* expansion of CAR-iT cells

A total of 2 × 10^5^ CD38 CAR-iT cells were co-cultured with a feeder mix containing 1 × 10^5^ EBV-LCLs (two donors) and 1 × 10^6^ PBMCs (three donors). PBMCs were irradiated with 25 Gy and EBV-LCLs were irradiated with 50 Gy. Cell mixture was suspended in medium containing RPMI-1640, 10% FBS, 100 U/mL penicillin, and 100 µg/mL streptomycin enriched with 1 µg/mL dox, 100 U/mL rhIL-2, 10 ng/mL rhIL-7, and 10 ng/mL IL-15. Medium along with cytokines and dox was replenished after 4 days. On the experiments assessing alloreactive potential, 1 µg/mL PHA-L was also added on the proliferation medium. In some experiments, CD38 CAR-iT cells were expanded in a feeder-mix consisting of irradiated EBV-LCLs (50 Gy) at a ratio 1:2.5 resuspended in RPMI proliferation medium as described above along with 1 µg/mL dox, 10 ng/mL rhIL-7, and 10 ng/mL rhIL-21. Fresh medium along with dox and cytokines was added after 4 days. For the maturation of CD19 CAR-iT cells, 150,000 effectors cells were co-cultured with 50,000 irradiated (50 Gy) 3T3-CD19 cells on Tdiff medium along with dox and cytokines for 7 days as described above. To assess the proliferation potential, mature CD19 CAR-iT cells were expanded on 3T3-CD19^+^ at E:T 3:1 on proliferation medium along with 1 µg/mL dox, 100 U/mL rhIL-2, 10 ng/mL rhIL-7, and 10 ng/mL IL-15 for 7 days.

For assessment of proliferation potential, CAR-iT cells were stained with 1 μμ Cell Trace Far Red (Thermo Fisher Scientific, Cat#C34564) according to manufacturer’s guidelines. Proliferation capacity was assessed after 1 week of stimulation with the allogeneic feeder mix as described above and evaluated by flow cytometry.

Data were further analyzed with FCS Express v7.

### Generation of primary CAR-T cells

Primary CAR-T cells were generated as previously described (39). Healthy donor PBMCs were stimulated with 1 µg/mL PHA-L at a concentration of 3 × 10^6^/well for 48 h. Activated T cells were transduced to express a CD38-28ζ CAR with γ-retroviral particles produced by a stable virus producer cell line 293Vec-RD114 (BioVec Pharma Inc.). TRE-mock T cells were generated by retroviral transduction with mock-LNGFR and TET viruses as previously described (19) Cell mixture along with 4 µg/mL polybrene (Sigma-Aldrich, Cat#107689) was spinoculated at 1500*g* for 1 h in RT followed by a second transduction step with fresh virus the day after. Twenty-four hours after the second transduction, two-thirds of the medium were replaced with fresh RPMI-1640 medium along with 10% FCS, 100 U/mL penicillin, 100 µg/mL streptomycin, and 10 ng/mL rhIL-7.

### Bioluminescence-based cytotoxicity assay

CAR-iT cells, treated with dox for 48 h to allow CAR expression or expanded after 1 week in feeder mix, were incubated with 10,000 Luc-GFP UM9 cells in different effector-to-target ratios. Cytotoxicity was determined after 16 h as the fraction of surviving UM9 cells based on bioluminescence emission after the addition of 125 mg/mL D-luciferin (Promega, Cat#E1605) for 30 min. Bioluminescence signal was quantified with a GloMax 96 Microplate Luminometer (Promega), and the percent lysis was measured based on the formula: 1 − (BLI signal in treated wells/BLI signal in untreated wells) × 100%.

To test their immunogenic potential, engineered T-iPSC were stably transduced with a lentiviral vector encoding Luciferase (Luc) and a puromycin selection marker (puro) [pRRL-cPPT-CMV-Luc2-IRES-GFP-PRE-SIN, after replacing GFP with a puromycin]. Luc-T-iPSC were seeded on Cultrex BME-coated plates cultured with mTESR medium supplemented with 500 U/mL recombinant human interferon-gamma (rhIFN-γ) (Peprotech, Cat#300-02) for 48–72 h to induce HLA class I upregulation. T-iPSC clones were then co-cultured either with allo-HLA-A*02 T cells or with NK cells for 24 h and bioluminescence signal was quantified as described above.

### Flow cytometry-based cytotoxicity assay

Allo-HLA-A*02 T cells were co-cultured with TCRα^−/−^β2m^+/+^and TCRα^−/−^β2m^−/−^HLA-E^+^ CD4^+^CD8αβ^+^ iDP thymocytes at serial dilutions for 24 h. To test the immunogenicity against NK cells, freshly isolated and expanded NKG2A^+^ NK cells were co-cultured with TCRα^+/+^β2m^−/−^ and TCRα^−/−^β2m^−/−^HLA-E^+^ CD4^+^CD8αβ^+^ iDP thymocytes at a ratio 9:1 for 24 h. Absolute numbers were calculated by using Flow-Count Fluorospheres (Beckman Coulter, Cat#7547053) as a reference. Percentage cell lysis was calculated as: % lysis = 1 − ((# viable target cells in treated wells/# of beads)/(# viable target cells in untreated wells/# of beads)) × 100%.

### Cytokine measurement

To measure the levels of secreted cytokines, supernatants from co-cultures of CAR-iT against UM9 were collected after 24 h. Cytokine amount was determined using the Cytometric Bead Array (CBA) Human Th1/Th2/Th17 Kit (BD Biosciences, Cat#560484) following the manufacturer’s guidelines.

### Immunophenotyping

The following conjugated antibodies were used for flow cytometry analysis: CD7-PE/Cy7 (CD7-6B7), 1:100; CD8α PE/Cy7 (HIT8a), 1:100 purchased from Sony Biotechnology; CD3-FITC (SK7), 1:50; CD3-BUV395 (UCHT1), 1:100; CD3-BUV737 (UCHT1), 1:100; CD4-BV650 (L200), 1:100; CD4-BUV395 (SK3), 1:100; CD8β-APC (2ST8.5H7), 1:25; CD38-HV450 (HB7), 1:20; CD45RA-BUV737 (HI100), 1:100; CD56-BUV737 (NCAM16.2), 1:100 purchased from BD Biosciences; CD5-PerCP (UCHT2),1:50; CD8α-APC/Cy7 (Hit8a), 1:50; CD27-BV785 (O323), 1:100; CD28-BV650 (CD28.2), 1:100; CD45RA-BV421 (HI100), 1:100; CD56-BV785 (5.1H11), 1:100; CD62L-PE/Cy7 (DREG-56), 1:100; CD159a (NKG2A)-APC (S19004C), 1:20; CD197 [CCR7 (G043H7)]-BV650, 1:50; HLA-ABC-PE/Cy7 (W6/32), 1:100; HLA-E-APC (3D12), 1:50; Zombie Aqua Fixable Viability Kit, 1:500; purchased from BioLegend; CD56-PE/Cy7 (N901) 1:100 purchased from Beckman Coulter; TCRαβ-APC (IP26), 1:50; TCRαβ-SuperBright780 (IP26), 1:100; LIVE/DEAD™ Fixable Near-IR Dead Cell Stain Kit, 1:1,000; Fixable Viability Dye eFluor™ 455UV, 1:500 purchased from Thermo Fisher Scientific; CD5-RPE (DK23), 1:50 purchased from Dako; and F(ab′)_2_ Fragment Goat Anti-Human IgG Fcγ fragment specific-AF647, 1:100 purchased from Jackson ImmunoResearch. GFP, mCherry, and BFP expression was measured in the FITC, PE-CF594, and DAPI channels, respectively. Sample readout was performed in LSRFortessa cytometer (BD Biosciences), and results were analyzed by using the FCS Express Flow Cytometry Version 6 software. Cell sorting was performed in in a BD FACSAria™ III Cell Sorter. For vendors and catalogue numbers, refer to **Supplementary Table S1**.

### *In vivo* xenograft studies

Hybrid scaffolds were subcutaneously implanted in mice, each consisting of three 2- to 3-mm^3^ biphasic calcium phosphate particles, coated *in vitro* with human BM mesenchymal stromal cells (BM-MSC; 2 × 10^5^ cells/scaffold). Eight to twelve weeks after implantation, mice were injected with Busulfan intraperitoneally [(18 mg/kg in 500 μL) (day −5), Teva], and the next day, they were intra-scaffold injected with luciferase-transduced multiple myeloma cells (0.5 × 10^6^ UM9 per scaffold) (day −4). Four days after injection of tumor cells (day 0), mice received indCD38CAR-iT cells (0.5 × 10^6^ cells/mice, *n* = 3 mice, 1 mouse did not survive anesthesia of day 0) intravenously or PBS (*n* = 4 mice). From day 0 until the end of the experiment, doxycycline (5 mg/kg) was intraperitoneally injected, at specific groups, three times per week. rhIL-15 was injected intraperitoneally (5 μg) from day 0 until day 10, every 2 days. rhIL-2 was injected intraperitoneally (50,000 U) every day from day 0 until day 10. Tumor growth was monitored by weekly bioluminescence (BLI) measurements using a PhotonIMAGER (Biospace Lab) (intraperitoneal injection of 100 μL of D-luciferin). To induce anesthesia, mice were subjected to isoflurane inhalation, 3% for initial induction and 1.5% for subsequent anesthesia. Upon reaching the Human Endpoint as established by the CCD, mice were euthanized by inhalation of 20% CO_2_.

### Statistical analysis

Data analysis and visualization were performed using GraphPad Prism v9.5.1 software. No pre-specified effect size was used to determine sample sizes. Graphs represent individual values ± standard error of the mean (SEM) for biological replicates or standard deviation (SD) for technical replicates. Normal data distribution was calculated by Shapiro-Wilk test. The statistical tests that were used to calculate the *p*-values are described in the relevant figure legends. Differences were considered significant at *p* < 0.05 and *p*-values are denoted with asterisks as follows: *p < 0.05, **p < 0.01, ***p < 0.001, ****p < 0.0001, ns, not significant.

## Data Availability

The original contributions presented in the study are included in the article/**Supplementary Material**. Further inquiries can be directed to the corresponding author.

## Supplementary material

Supplementary Figure S1 Original PCR gel related to **Figure 1B**.

Supplementary Figure S2 Original PCR gel related to **Figure 1C**.

Supplementary Figure S3 Original Southern Blot related to **Figure 1D**. **(A)** Verification for the specific integration of the GFP donor cassette into the *TRAC* locus **(B)** Target-specific confirmation for the integration of the mCherry donor template into the *TRAC* locus.

Supplementary Figure S4 Original immunoblot related to **Figure 1F**.

Supplementary Figure S5 Karyotypic analysis of WT and *TCRα*^-/-^ indCAR T-iPSC. Both the parental and the edited T-iPSC lines had a normal 46, XY karyotype.

Supplementary Figure S6 Immunophenotypic profile of TCRneg DP and indCD38CAR-iT cells. **(A)** Representative plots for T cell markers iDP thymocytes derived from clone GFP1. **(B)** Flow cytometry analysis of GFP1 TCR^neg^ CD8αβ^+^ indCD38CAR-iT cells after addition of dox for 48h. **(C)** Representative plot for CD38 expression at CD4^+^CD8αβ^+^ iDP thymocyte and CAR-iT CD8 SP stage derived from clone GFP9. **(D)** Relative fratricide upon CD38 engagement compared to iDP cells (n=3 independent differentiations). **(E)** Immunophenotype TCR^neg^ indCD19CAR-iT without dox (iDP stage), after dox without CD19 engagement and after dox addition upon CD19 encounter. of **(F)** Immunophenotyping of TCR^neg^ indCD38CAR-iT compared to primary CD8^+^ CD38CAR-T cells after 5 days of dox withdrawal or second transduction hit respectively. **(G)** RT-PCR for CAR expression on GFP9-derived cells at iDP, CD8 SP after dox and CD8 SP after removal of dox stage. **(H)** Flow cytometry analysis for surface expression kinetics of CD38 CAR. Grey histograms represent CAR expression on DP thymocytes without dox and red ones on CAR-iT after dox addition. **(I)** Expansion potential after weekly stimulations of CD38CAR-iT (clone 1.5 GFP4, n=1) **(J)** Proliferation capacity of CD19CAR-iT cells after weekly rechallenges (clone 1.5 19 G8, n=1).

Supplementary Figure S7 Original PCR gel related to **Figure S6G**.

Supplementary Figure S8 Validation of β2m locus editing and insertion of the HLA-E SCT expression-cassette. **(A)** PCR strategy for confirmation of successful integration for HLA-E SCT template into the *β2m* locus. Arrows show the amplified areas. Successful insertion creates a fragment of 1765 bp whereas the wild type generates a band of 2019 bp. **(B)** Representative flow cytometry plots for BFP, HLA class I (HLA-ABC) and HLA-E expression on GFP9BFP2.3 TCRα^-/-^ β2m^-/-^ HLA-E^+^ indCAR-T-iPSCs compared to the WT. **(C)** Karyotypic analysis of *TCRα*^-/^- *β2m*^-/^- HLA-E^+^ indCAR and *β2m*^-/^- HLA-E^+^ T-iPSC.

Supplementary Figure S9 Original non-targeted PCR gel related to **Figure S8B**.

Supplementary Figure S10 Original Southern Blot related to **Figure 4D**.

Supplementary Figure S11 Use of DL4 is needed for successful T cell development upon lack of B2M. **(A)** Representative flow cytometry plots of CD4 and CD8 expression in the presence or absence of B2M expression after co-culture with either OP9-DL1 or OP9-DL4 feeder cells for 25 days. **(B)** Immunophenotypic analysis TCR^neg^ B2M^neg^ indCD38CAR-iT after 5 days of dox withdrawal. **(C)** Histograms of surface CD38 CAR expression on TCR^neg^ B2M^neg^ indCD38CAR-iT cells upon dox withdrawal. Grey peaks display show CAR expression on no dox-treated iDP thymocytes and blue ones on CAR-iT after dox withdrawal.
