# Supplementary material for: Engineering of induced pluripotent stem cells for the efficient development of non-alloreactive, hypoimmunogenic CD8αβ CAR-T cells
Source: Front Immunol. 2026 Feb 20;17:1757174. doi: 10.3389/fimmu.2026.1757174 (PMC12963348; doi:10.3389/fimmu.2026.1757174)
Supplement: Supplementary file 1 [file Table1.docx]

Table S1. List of antibodies used in the present study

| Antibodies | Vendor | Cat No./ RRID |
| --- | --- | --- |
| Anti-human CD3 FITC | BD Biosciences | Cat#345763; RRID:AB_2811220 |
| Anti-human CD3 BUV395 | BD Biosciences | Cat#563546; RRID:AB_2744387 |
| Anti-human CD3 BUV737 | BD Biosciences | Cat#612750; RRID:AB_2870081 |
| Anti-human CD4 BV650 | BD Biosciences | Cat#563737; RRID:AB_2687486 |
| Anti-human CD4 BUV395 | BD Biosciences | Cat#563550; RRID:AB_2738273 |
| Anti-human CD5 PerCP | Biolegend | Cat#300617; RRID:AB_893378 |
| Anti-human CD7 PE/Cy7 | Sony Biotechnology | Cat#2315570; RRID:AB_2936333 |
| Anti-human CD8α APC/Cy7 | Biolegend | Cat#300926; RRID:AB_10613636 |
| Anti-human CD8α PE/Cy7 | Sony Biotechnology | Cat#2104565; RRID:AB_2936334 |
| Anti-human CD8β APC | BD Biosciences | Cat#641058; RRID:AB_1645723 |
| Anti-human CD27 BV785 | Biolegend | Cat#302832; RRID:AB_2562674 |
| Anti-human CD28 BV650 | Biolegend | Cat#302946; RRID:AB_2616855 |
| Anti-human CD38 HV450 | BD Biosciences | Cat#646851; RRID:AB_1937282 |
| Anti-human CD54RA BV421 | Biolegend | Cat#304130; RRID:AB_10965547 |
| Anti-human CD56 PE/Cy7 | Beckman Coulter | Cat#A21692; RRID:AB_2892144 |
| Anti-human CD56 BV785 | Biolegend | Cat#362550; RRID:AB_2566059 |
| Anti-human CD56 BUV737 | BD Biosciences | Cat#612766; RRID:AB_2813880 |
| Anti-human CD62L PE/Cy7 | Biolegend | Cat#304822; RRID:AB_830801 |
| Anti-human CD159a (NKG2A) APC | Biolegend | Cat#375107; RRID:AB_2888862 |
| Anti-human CD197 (CCR7) BV650 | Biolegend | Cat# 353234; RRID:AB_2563867 |
| Anti-human CD247 | BD Biosciences | Cat#556366; RRID:AB_396389 |
| Anti-human HLA-A,B,C PE/Cy7 | Biolegend | Cat#311430; RRID:AB_2561617 |
| Anti-human HLA-E APC | Biolegend | Cat#342605; RRID:AB_2565260 |
| Anti-human TCRαβ APC | eBioscience | Cat#17-9986-42; RRID:AB_10597896 |
| Anti-human TCRαβ SuperBright 780 | eBioscience | Cat#78-9986-42; RRID:AB_2735078 |
| Anti-Human IgG, F(ab')₂ fragment specific Alexa Fluor® 647 | Jackson Immunoresearch | Cat# 109-606-006; RRID:AB_2337893 |
| Anti-human actin | Sigma-Aldrich | Cat#MAB1501R; RRID:AB_2223041 |
| Anti-mouse IgG, HRP-linked Antibody | Cell Signaling Technology | Cat#7076; RRID:AB_330924 |
