# Supplementary figures and images for: Engineering of induced pluripotent stem cells for the efficient development of non-alloreactive, hypoimmunogenic CD8αβ CAR-T cells
Source: Front Immunol. 2026 Feb 20;17:1757174. doi: 10.3389/fimmu.2026.1757174 (PMC12963348; doi:10.3389/fimmu.2026.1757174)

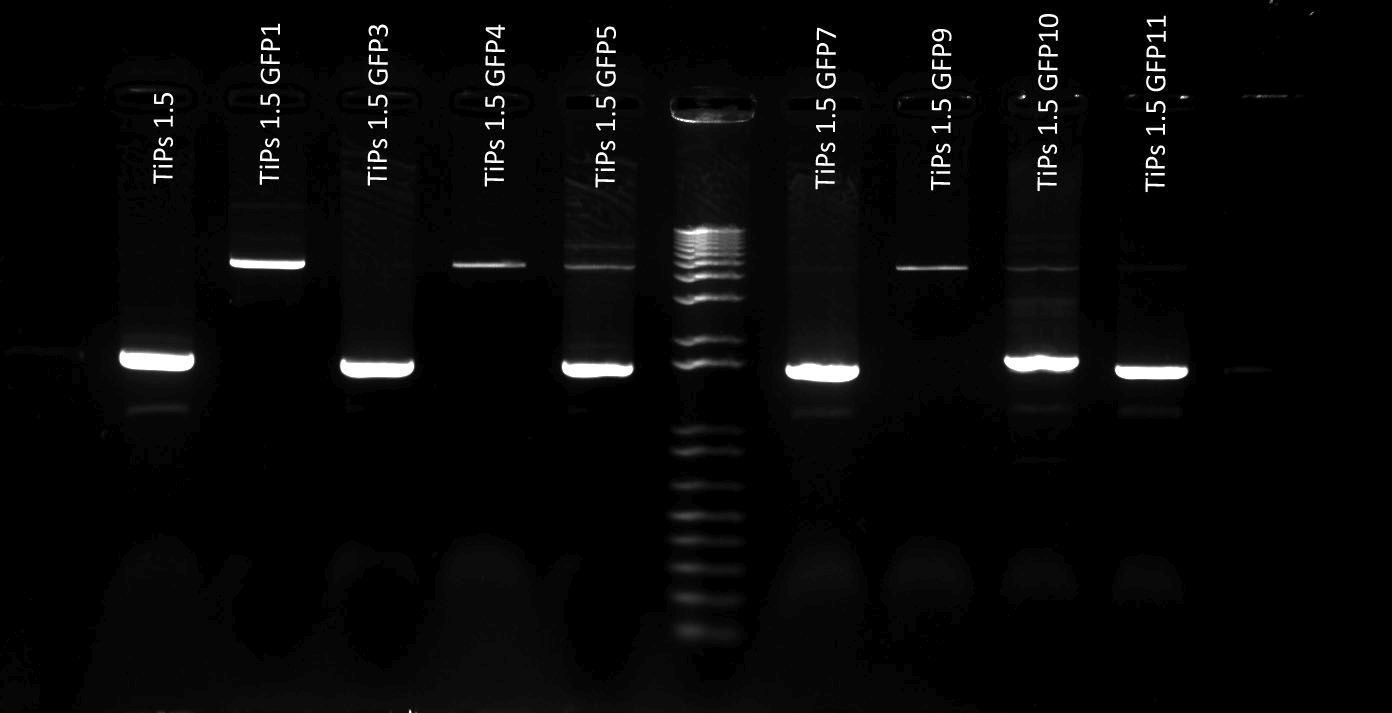

Supplement: Supplementary Figure S1 — Original PCR gel related to Figure 1B. [file Image1.jpeg]

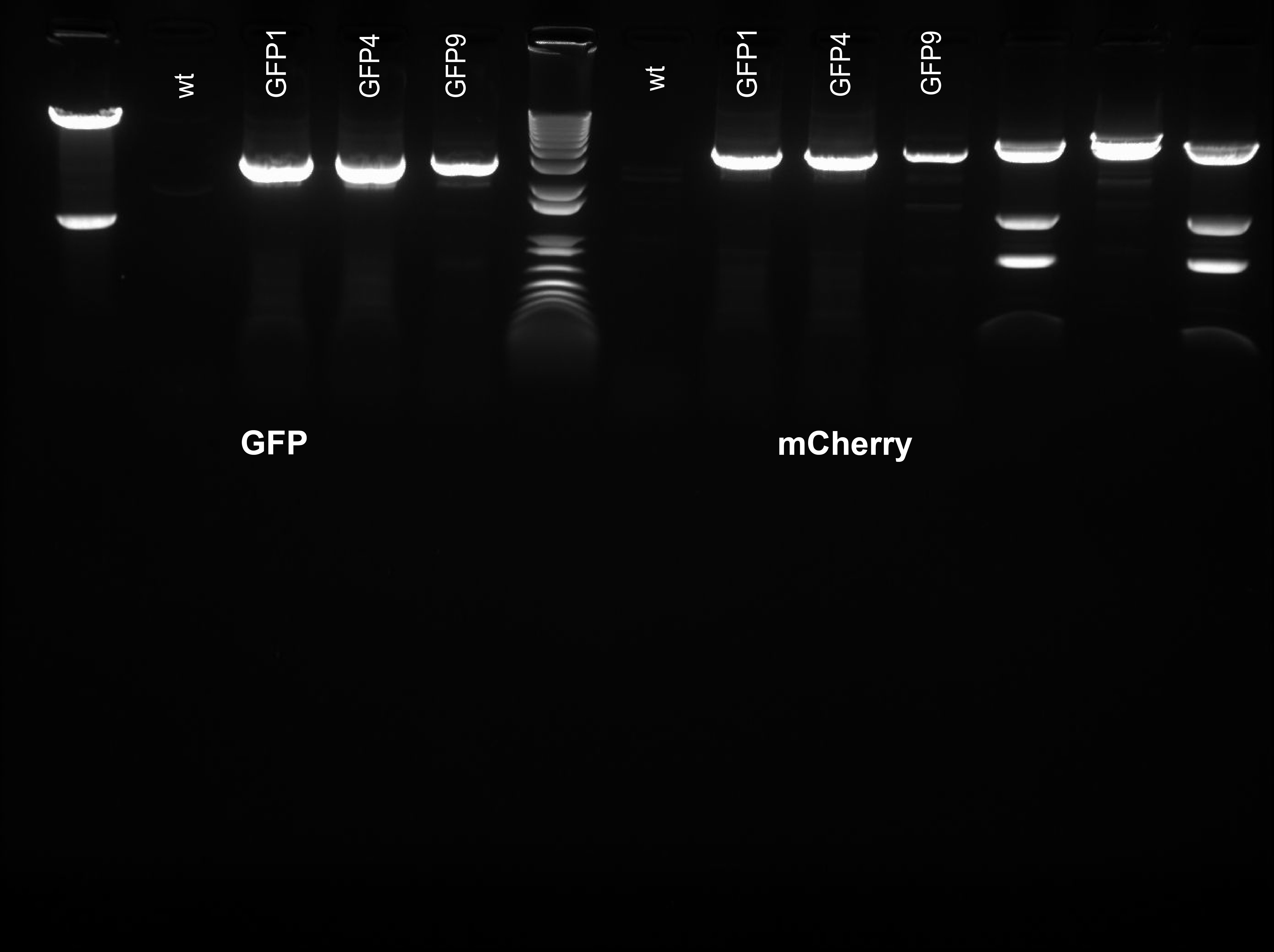

Supplement: Supplementary Figure S2 — Original PCR gel related to Figure 1C. [file Image2.jpeg]

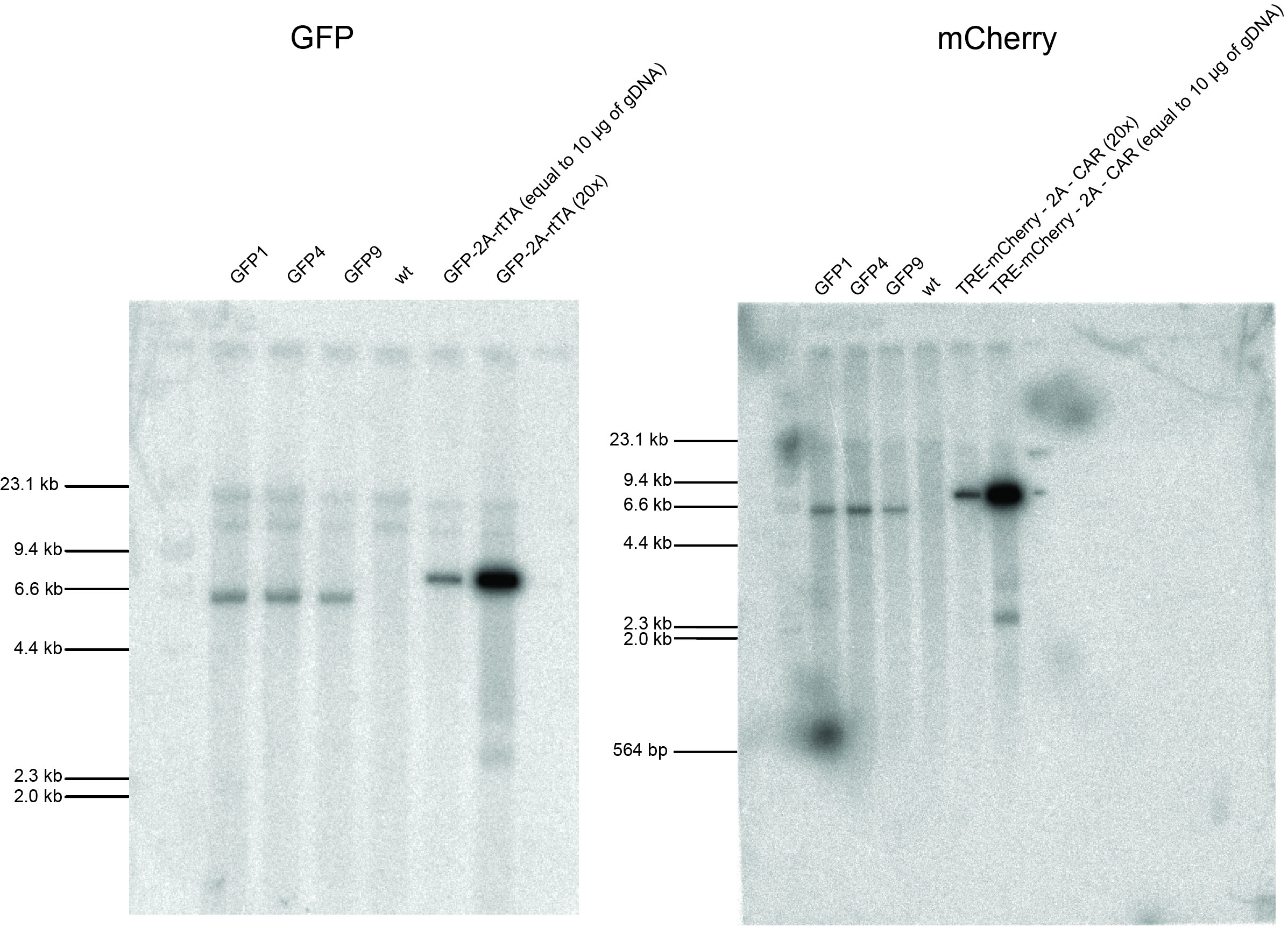

Supplement: Supplementary Figure S3 — Original Southern Blot related to Figure 1D. (A) Verification for the specific integration of the GFP donor cassette into the TRAC locus (B) Target-specific confirmation for the integration of the mCherry donor template into the TRAC locus. [file Image3.jpeg]

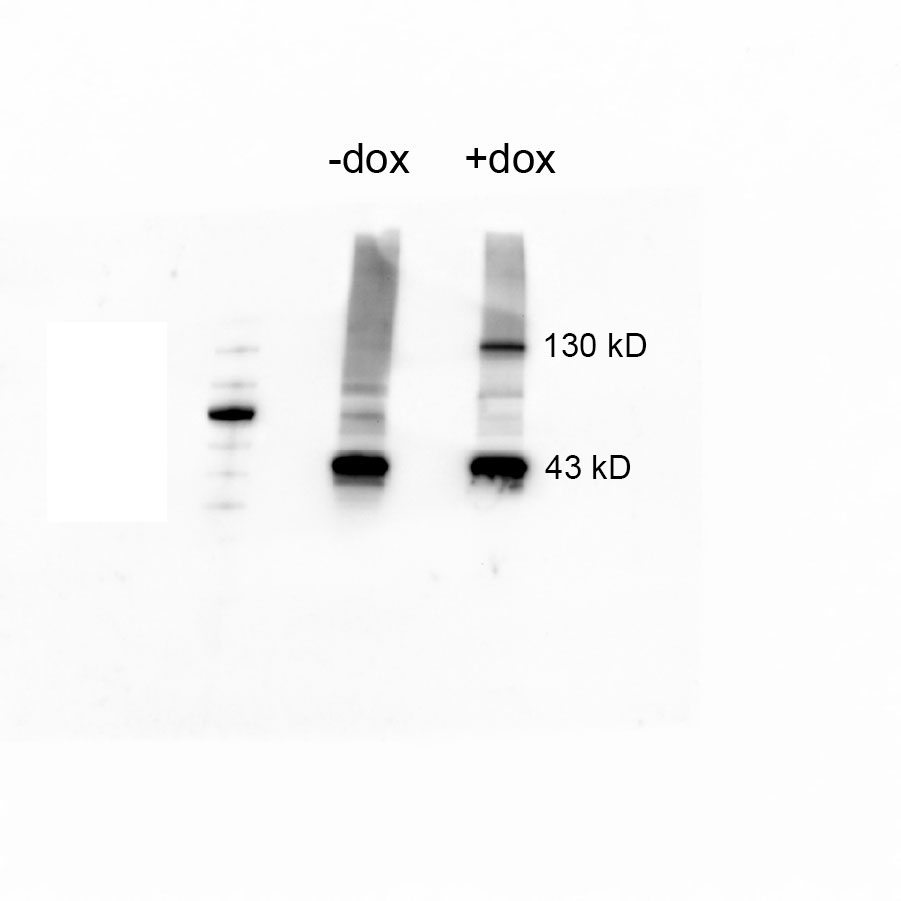

Supplement: Supplementary Figure S4 — Original immunoblot related to Figure 1F. [file Image4.jpeg]

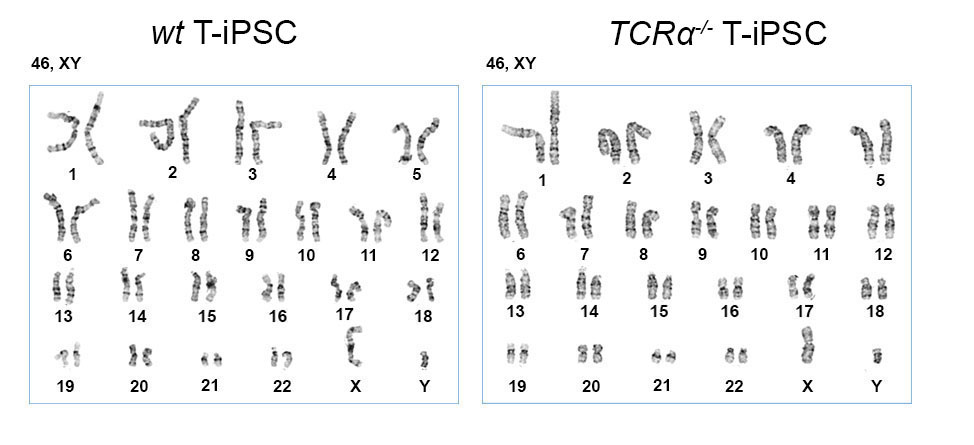

Supplement: Supplementary Figure S5 — Karyotypic analysis of WT and TCRα-/- indCAR T-iPSC. Both the parental and the edited T-iPSC lines had a normal 46, XY karyotype. [file Image5.jpeg]

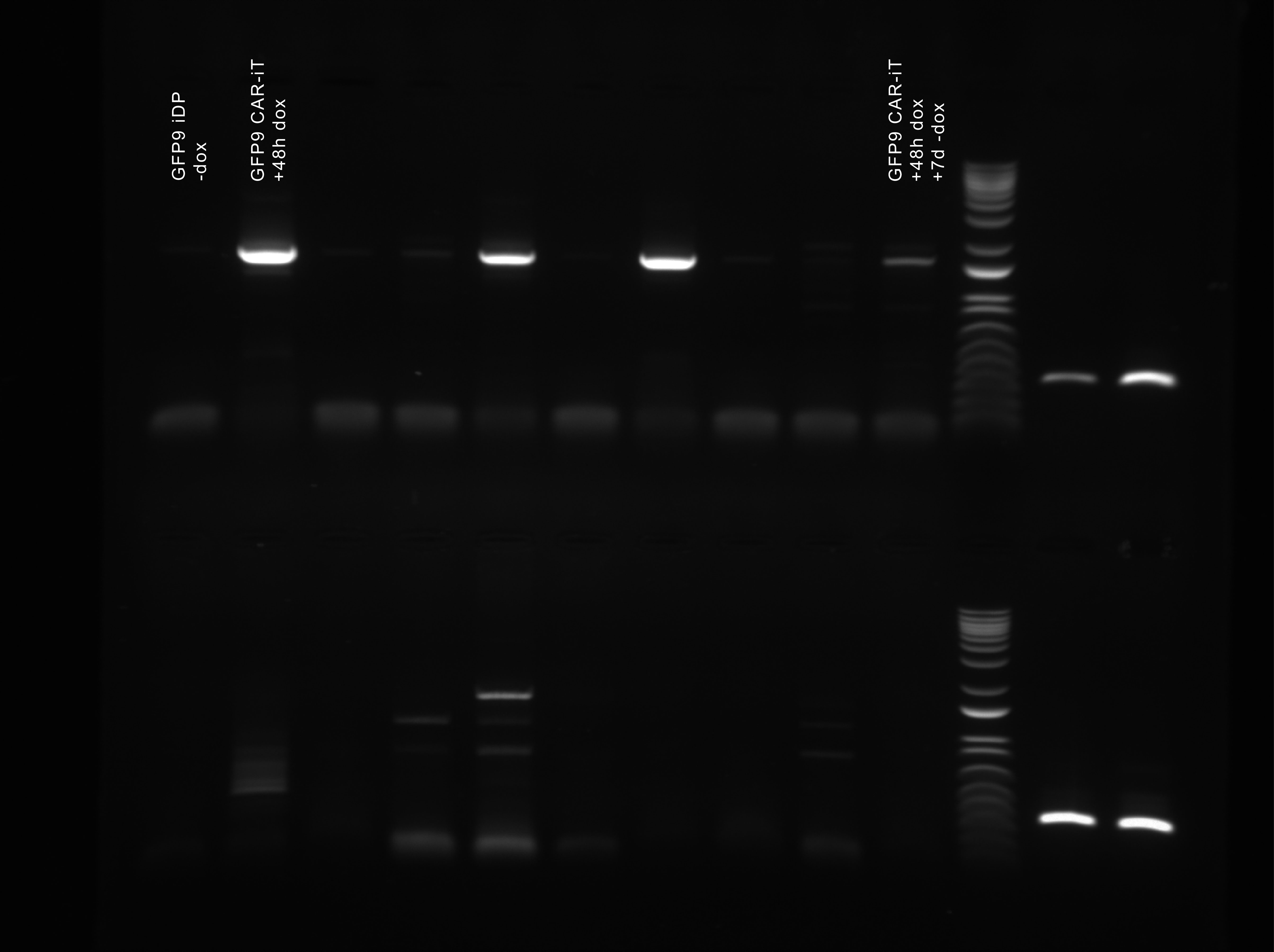

Supplement: Supplementary Figure S7 — Original PCR gel related to Figure S6G. [file Image7.jpeg]

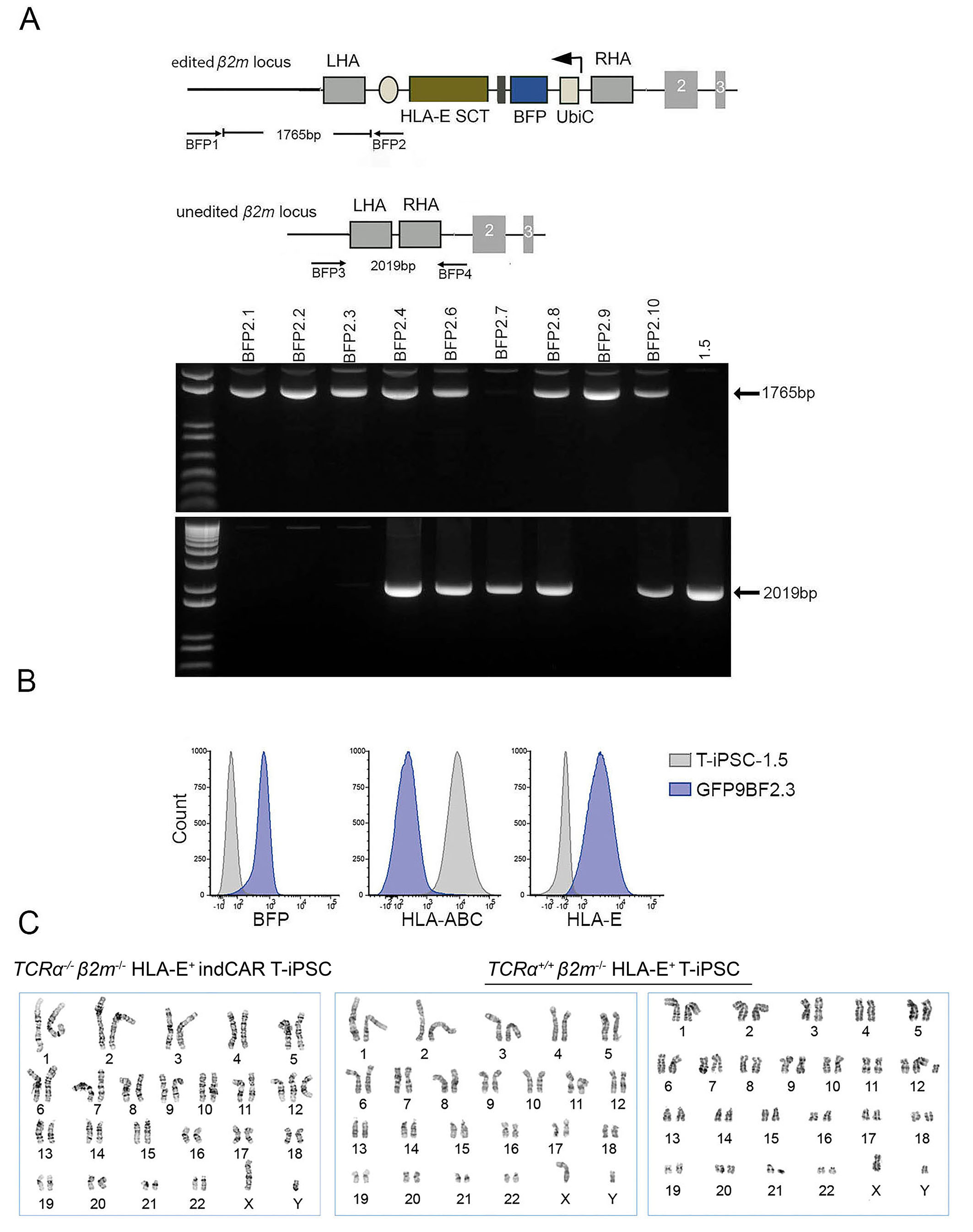

Supplement: Supplementary Figure S8 — Validation of β2m locus editing and insertion of the HLA-E SCT expression-cassette. (A) PCR strategy for confirmation of successful integration for HLA-E SCT template into the β2m locus. Arrows show the amplified areas. Successful insertion creates a fragment of 1765 bp whereas the wild type generates a band of 2019 bp. (B) Representative flow cytometry plots for BFP, HLA class I (HLA-ABC) and HLA-E expression on GFP9BFP2.3 TCRα-/- β2m-/- HLA-E+ indCAR-T-iPSCs compared to the WT. (C) Karyotypic analysis of TCRα-/- β2m-/- HLA-E+ indCAR and β2m-/- HLA-E+ T-iPSC. [file Image8.jpeg]

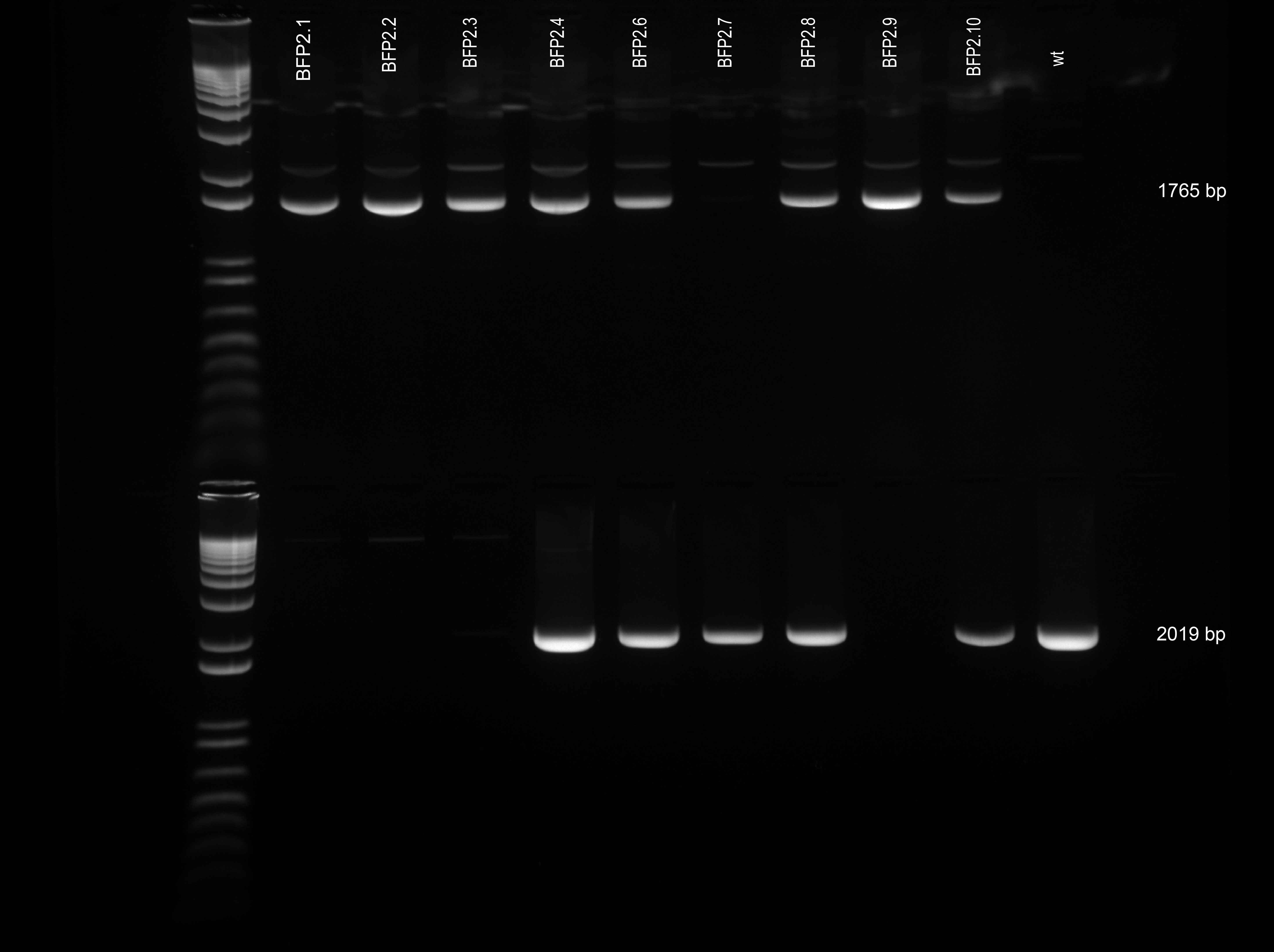

Supplement: Supplementary Figure S9 — Original non-targeted PCR gel related to Figure S8B. [file Image9.jpeg]

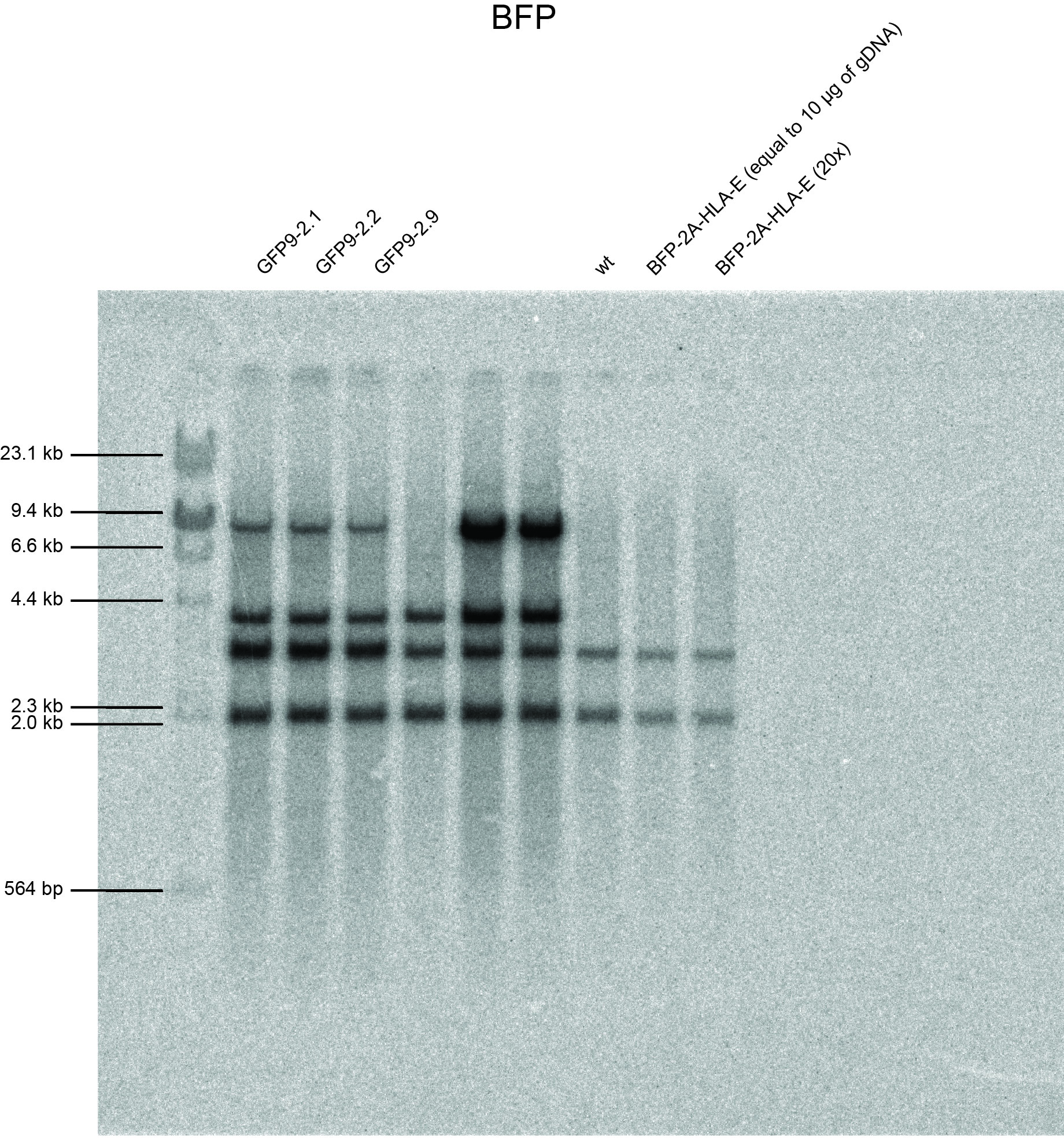

Supplement: Supplementary Figure S10 — Original Southern Blot related to Figure 4D. [file Image10.jpeg]

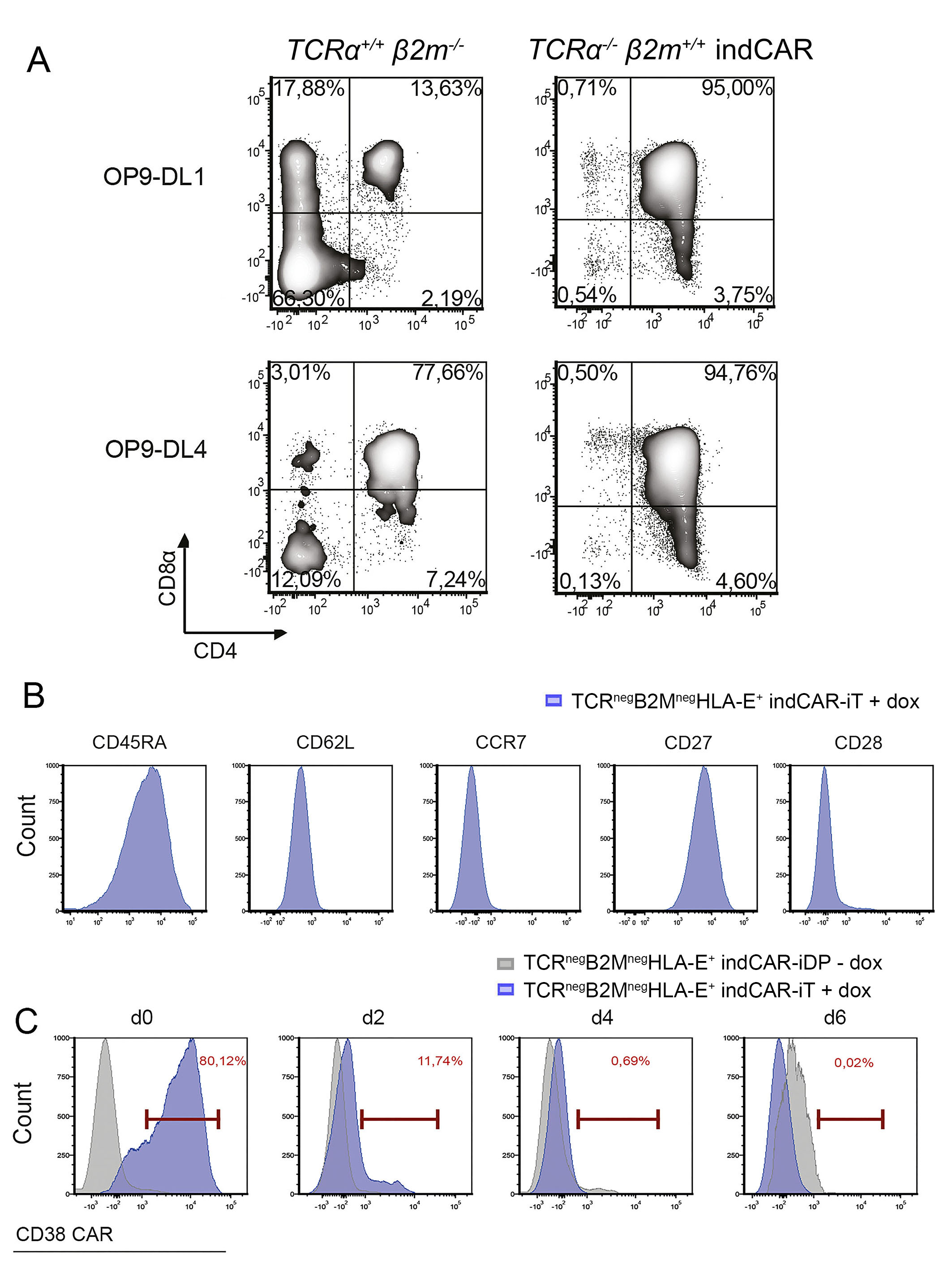

Supplement: Supplementary Figure S11 — Use of DL4 is needed for successful T cell development upon lack of B2M. (A) Representative flow cytometry plots of CD4 and CD8 expression in the presence or absence of B2M expression after co-culture with either OP9-DL1 or OP9-DL4 feeder cells for 25 days. (B) Immunophenotypic analysis TCRneg B2Mneg indCD38CAR-iT after 5 days of dox withdrawal. (C) Histograms of surface CD38 CAR expression on TCRneg B2Mneg indCD38CAR-iT cells upon dox withdrawal. Grey peaks display show CAR expression on no dox-treated iDP thymocytes and blue ones on CAR-iT after dox withdrawal. [file Image11.jpeg]
